# A multi-omic analysis reveals the regulatory role of CD180 during the response of macrophages to *Borrelia burgdorferi*

**DOI:** 10.1038/s41426-017-0018-5

**Published:** 2018-03-07

**Authors:** Ana Carreras-González, Nicolás Navasa, Itziar Martín-Ruiz, José Luis Lavín, Mikel Azkargorta, Estíbaliz Atondo, Diego Barriales, Nuria Macías-Cámara, Miguel Angel Pascual-Itoiz, Leticia Sampedro, Julen Tomás-Cortázar, Ainize Peña-Cearra, Aize Pellón, Rafael Prados-Rosales, Leticia Abecia, Félix Elortza, Ana M. Aransay, Héctor Rodríguez, Juan Anguita

**Affiliations:** 1 Macrophage and Tick Vaccine Laboratory, CIC bioGUNE, 48160 Derio, Bizkaia Spain; 2 Genome Analysis Platform, CIC bioGUNE, 48160 Derio, Bizkaia Spain; 3 Proteomics Platform, CIC bioGUNE, 48160 Derio, Bizkaia Spain; 4 Carlos III Networked Proteomics Platform (ProteoRed-ISCIII), Universidad Autónoma de Madrid, 28049 Cantoblanco, Madrid, Spain; 5 Universidad del País Vasco/Euskal Herriko Unibertsitatea, 48940 Leioa, Bizkaia Spain; 6 Centro de Investigación Biomédica en Red de enfermedades hepáticas y digestivas (CIBERehd), Instituto de Salud Carlos III, 28029 Madrid, Spain; 7 Ikerbasque, Basque Foundation for Science, 48011 Bilbao, Bizkaia Spain

## Abstract

Macrophages are cells of the innate immune system with the ability to phagocytose and induce a global pattern of responses that depend on several signaling pathways. We have determined the biosignature of murine bone marrow-derived macrophages and human blood monocytes using transcriptomic and proteomic approaches. We identified a common pattern of genes that are transcriptionally regulated and overall indicate that the response to *B. burgdorferi* involves the interaction of spirochetal antigens with several inflammatory pathways corresponding to primary (triggered by pattern-recognition receptors) and secondary (induced by proinflammatory cytokines) responses. We also show that the Toll-like receptor family member CD180 is downregulated by the stimulation of macrophages, but not monocytes, with the spirochete. Silencing *Cd180* results in increased phagocytosis while tempering the production of the proinflammatory cytokine TNF. *Cd180*-silenced cells produce increased levels of *Itgam* and surface CD11b, suggesting that the regulation of CD180 by the spirochete initiates a cascade that increases CR3-mediated phagocytosis of the bacterium while repressing the consequent inflammatory response.

## Introduction

Innate immune responses constitute the first host defense mechanism against infection. Phagocytic cells, particularly macrophages and dendritic cells, recognize and internalize bacteria by phagocytosis^1^. In addition to the elimination of invading pathogens, the internalization of microorganisms via phagocytosis is the starting point for several downstream signaling pathways, including proinflammatory cascades, as well as those associated with antigen presentation and the activation of cells involved in acquired immune responses. Thus, phagocytosis has critical consequences for the overall response to an invading pathogen, and its coupling to inflammation must be tightly regulated. Innate immune responses to pathogens occur via the activation of different families of receptors, which collectively are referred to as pattern-recognition receptors (PRRs). The best-known examples of PRRs are the families of Toll-like receptors (TLRs), C-type lectin receptors, NOD-like receptors, and RIG-I-like receptors^2^. These molecules respond to specific pathogen components^3^ and are ideal candidates as couplers of phagocytosis to inflammation since they are recruited to phagosomes.

Lyme borreliosis, which is caused by members of the spirochete family from the genus *Borrelia*, is the most common arthropod-borne infection in Europe, with at least 65,000 documented cases every year^4^, and in North America^5,6^, where an estimated 300,000 cases occur annually^7^. Symptoms associated with Lyme borreliosis may be debilitating and long-lasting despite appropriate antibiotic treatment. Local inflammatory responses during early infection often result in the appearance of a skin rash (erythema migrans) at the inoculation site. The initial skin inflammatory reaction can be accompanied by other symptoms such as fever, headache, malaise, myalgia, and/or arthralgia^8^. The spirochete disseminates hematogenously and colonizes different tissues and/or organs, inducing a variety of inflammatory symptoms including conduction system abnormalities, meningitis, and acute arthritis^9,10^. Some untreated individuals develop persistent forms of the disease that are normally associated with the prolonged infection with the spirochete^11^. Extensive research has been performed in the last several years to identify the receptors and signals related to the internalization and immune responses to *B. burgdorferi*. TLRs have an important role in sensing spirochete constituents and triggering immune responses^12^. The involvement of other surface receptors such as CR3-CD14^13^, MARCO^14^, or uPAR^15^ in both internalization and macrophage-dependent immune modulation has also been recently described. The importance of the phagocytic process during infection with *B. burgdorferi* is twofold: first, it allows the control of spirochetal numbers during infection, especially in those organs susceptible to developing an inflammatory process, such as the heart, in which phagocytosis seems to outperform the elimination of the bacteria by circulating antibodies^16,17^; second, phagocytosis modulates both the quality and quantity of the inflammatory response by allowing the interaction of pathogen-associated molecular patterns (PAMPs) with their cognate PRRs upon degradation of the bacteria in phagolysosomal compartments^18–21^. Phagocytosis of *B. burgdorferi* by macrophages and other cell types is, therefore, probably one of the most important mechanisms of control of the bacteria in the mammalian host.

Large-scale analytical approaches to quantify gene expression (transcriptomics), proteins (proteomics), and metabolites (metabolomics) have emerged with a potential to advance the identification of biomarkers in early, disseminated and post-treatment disease stages^22^. These technologies may permit a definition of the disease stage and facilitate its early detection to improve diagnosis. Here we employed different omic techniques to study innate immune responses to the spirochete using both murine macrophages and human monocytes. Using RNAseq, we show the global transcriptional profile of primary murine macrophages after exposure to *B. burgdorferi*. Our results confirm the involvement of some immune traits that have been previously described during the activation of macrophages upon *B. burgdorferi* stimulation and reveal new genes that might be involved in these processes. We also found that these transcriptional traits are present in human monocytes stimulated with the spirochete, albeit with several differences that are probably species and/or cell type (monocytes vs. macrophages)-dependent. Finally, we use a proteomic analysis of *B. burgdorferi*-stimulated murine bone marrow-derived macrophages to further corroborate the transcriptomic findings and identify novel regulators of the response to the bacterium. In particular, we describe the regulation of CD180 in response to *B. burgdorferi*. Our results show that CD180 modulates both phagocytosis and inflammation in response to *B. burgdorferi* through the transcriptional repression of complement receptor 3 (CR3). Overall, our data define the specific expression traits in monocytes/macrophages in response to the spirochete, allowing the identification of components that modulate the specific interaction between the pathogen and innate immune cells.

## Materials and methods

### Mice

C57Bl/6 (B6) mice were purchased from Charles River Laboratories (Barcelona, Spain) and bred at CIC bioGUNE. All work performed with animals was approved by the competent authority (Diputación de Bizkaia) following European and Spanish directives. The CIC bioGUNE’s Animal Facility is accredited by AAALAC Intl.

### Bacteria

*B. burgdorferi* Bb914, a clone derived from strain 297 that contains a constitutively expressed GFP reporter stably inserted into cp26^23^, along with wild-type *B. burgdorferi* 297 were used for BMMs and RAW264.7 cells. *B. burgdorferi* clone 5A15 of strain B31 was used to stimulate human monocytes and monocyte-derived macrophages. Bacteria were grown in 5-ml tubes at 34 °C in BSK-H medium (Sigma Aldrich Quimica SL, Madrid, Spain). All stimulations were performed for a period of 16 h unless otherwise stated.

### Cell culture

The macrophage-like RAW264.7 cell line was maintained in DMEM (Lonza, Barcelona, Spain) supplemented with 10% FCS, 2.4 mM l-glutamine and 10% penicillin–streptomycin (Thermo Fisher Scientific, Waltham, MA). RAW264.7 cells were washed and resuspended in FCS– and penicillin–streptomycin-free DMEM 2 h before use.

Bone marrow-derived macrophages (BMMs) were generated from 8–12-week-old C57Bl/6 (B6) mice as described^16^. Bone marrow cells were collected from the femoral shafts and incubated in 100 mm × 15 mm Petri dishes (Thermo Fisher Scientific) for 8 days in DMEM supplemented with 10% FCS and 10% penicillin–streptomycin plus 30 ng/ml of M-CSF (Miltenyi Biotec, Bergisch Gladbach, GE). Following incubation, non-adherent cells were eliminated, and adherent macrophages were scraped, counted and seeded in 6-well tissue-culture plates for stimulation at a density of 10^6^ cells per ml. Macrophages were allowed to rest overnight prior to stimulation.

Lentiviral particles containing shRNA targeting *Clec4e* (Sigma Aldrich) and *Cd180* (OriGene Technologies, Rockville, MD) were produced as previously described^24^. Supernatants containing the virus were used to infect RAW264.7 cells, followed by incubation with puromycin at 3 µg/ml to generate stable lines. Cells containing the empty vector pLK0.1 were used as controls.

Human monocytes were purified from buffy coats of healthy blood donors by positive selection using a human CD14 purification kit (Miltenyi Biotec). Peripheral blood monocytic cells were isolated by Ficoll density centrifugation at 400x*g* for 30 min without brakes. The monocyte layer was recovered, washed, and processed according to the manufacturer’s protocol. Monocytes were allowed to rest for at least 4 h before stimulation. To obtain macrophages, purified human monocytes were incubated for 8 days in RPMI 1640 medium (Lonza) supplemented with 10% FCS, 2.4 mM l-glutamine, 10% penicillin–streptomycin and 30 ng/ml of human M-CSF (Miltenyi Biotec). The cells were rested overnight before stimulation. All human samples were obtained after approval by the Basque Country’s Ethics committee following the Helsinki convention. Donors signed an informed consent form and were anonymized to the authors.

### Phagocytosis assays

Phagocytosis assays were performed as previously described^13^. Experiments were performed in DMEM medium without serum or antibiotics. The day before the assay, the cells were seeded at a density of 1 × 10^6^ cells per ml. After 24 h, *B. burgdorferi* was added to the cells at a multiplicity of infection (m.o.i.) of 25 and incubated at 4 °C for 15 min followed by 37 °C for 1.5 h. The cells were then washed to eliminate surface bacteria and analyzed by flow cytometry or confocal microscopy.

### Confocal microscopy

Following incubation of the cells with bacteria, the cells were washed extensively, fixed in 3.7% paraformaldehyde for 7 min and then washed with PBS. The cells were then permeabilized with 0.1% Triton-X for 5 min and washed. After blocking non-specific binding with 5% BSA for 60 min, the cells were stained with rhodamine phalloidin for 10 min to visualize the actin cytoskeleton followed by DAPI for 10 min to stain the nuclei (Thermo Fisher Scientific), both at 37 °C. After extensive washing in PBS, the cells were mounted with Prolong Gold Anti-fade mounting reagent (Thermo Fisher Scientific). Photomicrographs were obtained using a Zeiss LSM 880 Confocal System.

### TNF ELISA

The levels of TNF produced by *B. burgdorferi* stimulation were determined by capture ELISA using the DuoSet II kit (R&D Systems, Minneapolis, MN) according to the manufacturer’s recommendations.

### RNA extraction

Total RNA was extracted using the NucleoSpin® RNA kit (Macherey-Nagel, Düren, GE). The quantity and quality of the RNAs were evaluated using the Qubit RNA Assay Kit (Thermo Fisher Scientific) and RNA Nano Chips in a 2100 Bioanalyzer (Agilent Technologies, Santa Clara, CA), respectively.

### RNAseq transcriptomics

Libraries for sequencing were prepared using the TruSeq RNA Sample Preparation Kit v2 (Illumina Inc., San Diego, CA) following the manufacturer’s instructions. Single-read 50-nt sequencing of pooled libraries was carried out in a HiScanSQ platform (Illumina Inc.). The data were generated from macrophages differentiated from three independent mice.

### Gene expression array

Total RNA (200 ng) was used to characterize gene expression with Illumina Human HT12 v4 BeadChips (GPL10558) containing 48,804 probes derived from Human RefSeq build 36.2. The cRNA synthesis, amplification, labeling and hybridization of the RNAs were performed following the Whole-Genome Gene Expression Direct Hybridization protocol (Illumina Inc.). cRNAs were then hybridized to the diverse gene-probes of the array, and the gene expression levels of the samples were detected using a HiScan scanner (Illumina Inc.). Raw data were extracted with GenomeStudio analysis software (Illumina Inc.) in the form of GenomeStudio’s Final Report (sample probe profile). The data were generated using purified monocytes from three donors.

### Data analysis

Quality control of the RNAseq sequenced samples was performed using FASTQC software (www.bioinformatics.babraham.ac.uk/projects/fastq). Reads were mapped against the whole mouse (*mm10*) reference genome by Tophat^25^ to account for splice junctions. The resulting BAM alignment files for the samples were the input for the differential expression (DE) analysis carried out by DESeq2^26^ to account for differentially expressed genes between *B. burgdorferi*-stimulated and unstimulated macrophages. Alignment files were taken as input to generate a table of read counts via R/Bioconductor package GenomicAlignments through the *sumarizeOvelaps* function in “union” mode for single reads/experiment. The number of uniquely mapped reads ranged from 23 to 25 × 10^6^ per sample.

For array data analysis, first, raw expression data were background-corrected, log_2_-transformed and quantile-normalized using the *lumi* R package^27^ available through the Bioconductor repository. Probes with a “detection *p*-value” lower than 0.01 in at least one sample were selected. For the detection of differentially expressed genes between *B. burgdorferi*-stimulated and unstimulated monocytes, a linear model was fitted to the probe data, and empirical Bayes moderated t-statistics were calculated using the *limma* package^28^ from Bioconductor. Only genes with a differential fold change (FC) >2 or <−2 and a *p*-value < 0.05 were considered differentially expressed.

GO enrichment was tested using the clusterProfiler^29^ Bioconductor package and the Panther Database^30^. Gene Ontology enrichment assessment was achieved according to GO^31^ and KEGG^32^ database terms. The data were also analyzed using QIAGEN’s Ingenuity^®^ Pathway Analysis (IPA, QIAGEN, Red Wood City, CA).

### Real-time RT-PCR

RNA was reverse-transcribed using M-MLV reverse transcriptase (Thermo Fisher Scientific). Real-time PCR was then performed using SYBR Green PCR Master Mix (Thermo Fisher Scientific) on a QuantStudio 6 real-time PCR System (Thermo Fisher Scientific). The fold induction of the genes was calculated using the 2^−ΔΔCt^ method relative to the reference genes, *Rpl19* (mouse) or *RNABP1* and *PLXNC1* (human). The primers used are listed in Supplementary Table S1.

### Label-free (LF) mass spectrometry proteomics analysis

Total protein from *B. burgdorferi*-stimulated and unstimulated BMMs (from three independent mice) was extracted using 3.5 M urea, 1 M thiourea, and 2% CHAPS. The samples were incubated for 30 min at RT under agitation and digested following the filter-aided sample preparation (FASP) protocol described by Wisniewski et al^33^ with minor modifications. Trypsin was added at a trypsin:protein ratio of 1:10, and the mixture was incubated overnight at 37 °C, dried in an RVC2 25 speedvac concentrator (Christ), and resuspended in 0.1% formic acid.

The equivalent of approximately 500 ng of each sample was analyzed by LC–MS LF analysis. Peptide separation was performed on a nanoACQUITY UPLC System (Waters, Cerdanyola del Vallès, Barcelona, Spain) connected on-line to an LTQ Orbitrap XL mass spectrometer (Thermo Fisher Scientific). An aliquot of each sample was loaded onto a Symmetry 300 C18 UPLC Trap column (180 µm × 20 mm, 5 µm -Waters-). The pre-column was connected to a BEH130 C18 column (75 μm × 200 mm, 1.7 μm (Waters) and equilibrated in 3% acetonitrile and 0.1% FA. Peptides were eluted directly into an LTQ Orbitrap XL mass spectrometer (Thermo Finnigan, Somerset, NJ) through a nanoelectrospray capillary source (Proxeon Biosystems, Thermo Fisher Scientific) at 300 nl/min and using a 120 min linear gradient of 3–50% acetonitrile. The mass spectrometer automatically switched between MS and MS/MS acquisition in DDA mode. Full MS scan survey spectra (*m*/*z* 400–2000) were acquired in the orbitrap with a mass resolution of 30000 at *m*/*z* 400. After each survey scan, the six most intense ions above 1000 counts were sequentially subjected to collision-induced dissociation (CID) in the linear ion trap. Precursors with charge states of 2 and 3 were specifically selected for CID. Peptides were excluded from further analysis during 60 s using the dynamic exclusion feature.

Progenesis LC–MS (version 2.0.5556.29015, Nonlinear Dynamics, Newcastle Upon Tyne, UK) was used for the LF differential protein expression analysis. One of the runs was used as the reference to which the precursor masses in all other samples were aligned. Only features comprising charges of 2+ and 3+ were selected. The raw abundances of each feature were automatically normalized and logarithmized against the reference run. Samples were grouped in accordance with the comparison being performed, and an ANOVA analysis was performed. A peak list containing the information for all the features was generated and exported to the Mascot search engine (Matrix Science Ltd., London, UK). This file was searched against a Uniprot/Swissprot database, and the list of identified peptides was imported back to Progenesis LC–MS. Protein quantification was performed based on the three most intense non-conflicting peptides (peptides occurring in only one protein), except for proteins with only two non-conflicting peptides. The significance of expression changes was tested at the protein level, and proteins with an absolute value of log_2_(fold change) ≥ 1 and ANOVA *p*-value ≤ 0.05 were selected for further analyses.

### Statistical analysis

The results are presented as the means ± SE (standard error). Significant differences between means were calculated with the Student’s t test. A *p*-value < 0.05 was considered significant. The Benjamini–Hochberg adjustment method was used for multiple hypothesis testing in DESeq2 (RNAseq) and *limma* (microarray).

### Data availability

The transcriptomic and microarray data are deposited under GEO accession number GSE103483. The proteomics data are deposited in ProteomeXchange under the accession number PXD008228.

## Results

### Global transcriptional response of murine BMMs to *B. burgdorferi* stimulation

**Fig. 1:**
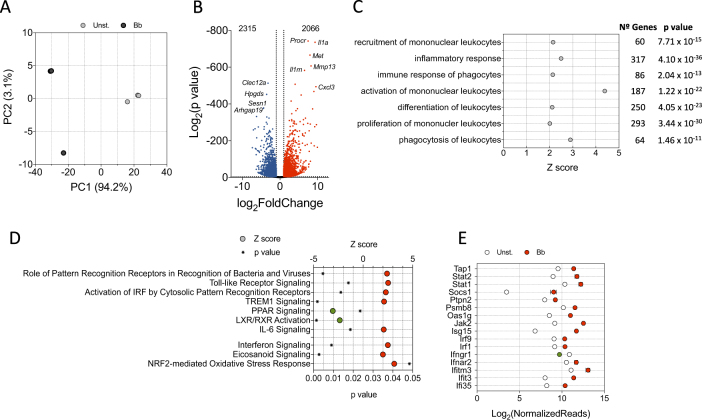
RNAseq analysis of murine BMMs stimulated with *B. burgdorferi*. **a** PCA of BMMs stimulated with *B. burgdorferi* (black circles) or left unstimulated (gray circles). **b** Volcano plot showing genes upregulated (red dots) or downregulated (blue dots) upon stimulation of BMMs with *B. burgdorferi*. Ten of the most regulated genes are identified. **c** Biological processes that are significantly regulated by the stimulation of BMMs with *B. burgdorferi*. The Z scores identify those processes that are differentially regulated (*Z* > 2). The number of genes corresponding to each process and the *p*-value obtained by IPA is also shown. **d** *Z* scores corresponding to genes regulated by *B. burgdorferi* in BMMs in response to specific PRRs and upstream regulators. *Z* scores >2 or <−2 were considered significant. **e** Genes that are differentially regulated by *B. burgdorferi* in BMMs (red circles = upregulated; green circle = downregulated) corresponding to the interferon signaling pathway according to IPA

To unveil main transcriptional traits involved in the response of macrophages to the spirochete, we performed RNAseq comparing non-stimulated with *B. burgdorferi*-stimulated BMM. Principal component analysis (PCA) (Fig. 1a) and sample distance matrix (Supplementary Figure S1) showed a clear transcriptional signature derived from BMM exposure to the spirochete. A total of 2066 genes were upregulated and 2315 were downregulated when applying cut-off values of twofold induction (absolute value of log_2_ ratio ≥ 1) and a p value of 0.05 (Fig. 1b). Representative upregulated and downregulated genes were validated by qRT-PCR (Supplementary Figure S2, see also Fig. 3d, f). We then performed an ingenuity pathway analysis (IPA) to obtain information about pathways that were triggered by *B. burgdorferi* exposure. As expected, the stimulation of BMMs with *B. burgdorferi* triggered a pattern of gene expression consistent with the recruitment, activation and proliferation of mononuclear leukocytes (Fig. 1c). Overall, a large number of genes that were regulated by *B. burgdorferi* (317 genes) were related to inflammatory responses, and a sizeable amount (64 genes) identified phagocytosis as a hallmark of the response (Fig. 1c).

The expression profile of macrophages exposed to the spirochete resembled the transcriptional fingerprint upon recognition of bacteria and viruses by PRRs, including Toll-like receptor signaling or activation of IRF by cytosolic PRRs (Fig. 1d). Furthermore, the expression profile significantly resembled TREM1 receptor signaling events (Fig. 1d) upon stimulation. This protein acts as an amplifier of monocyte inflammatory responses triggered by infections by stimulating the release of proinflammatory chemokines and cytokines. In contrast, signaling intermediates induced by PPAR, a well-recognized suppressor of inflammatory responses^34–36^, were significantly repressed upon stimulation with *B. burgdorferi* (Fig. 1d). We observed a significant overlap of the genes regulated by stimulation of BMMs with *B. burgdorferi* and those associated with gene expression dependent on LPS-, poly rI:C-RNA-, and NOD2 (Table 1). These data confirmed the complex nature of the interaction signature between *B. burgdorferi* and PRRs that depends on several receptors. Indeed, although significant overlapping was also found when comparing genes regulated by PMA_3_CSK_4_ or TLR2-induction with stimulated BMMs (Table 1), the percentage of genes that were shared among these conditions was <10%, indicating that the overall response to the spirochete is dependent on a larger array of signaling receptors.

**Table 1:** Selected upstream upregulators showing a significant overlap with the stimulation of BMM with *B. burgdorferi*^a^

Regulator	Log_2_ (fold induction)	*Z* score	*p* value	No. of molecules	Total (%)
Lipopolysaccharide		11.582	2.07E−97	527	931 (57)
Poly rI:C-RNA		8.706	1.49E−41	161	721 (22)
*Nod2*	3.373	5.265	2.61E−19	41	743 (6)
NFkB complex		6.403	2.07E−33	185	782 (24)
p38 MAPK		3.655	5.28E−19	110	759 (15)
*Tnf*	5.200	9.444	1.49E−68	474	837 (57)
*Il1b*	7.704	7.637	2.90E−55	288	823 (35)
*Il6*	7.634	4.026	2.96E−40	221	835 (27)
*Nos2*	6.355	3.853	1.01E−10	59	869 (7)
*Mif*	1.367	3.255	2.07E−07	36	751 (5)
*Ifnb1*	2.673	5.798	1.39E−42	123	622 (20)
*Il10ra*	−0.688	−7.569	3.23E+00	137	498 (28)
PAM_3_CSK_4_		4.754	1.47E−17	51	587 (9)
*Tlr2*	1.556	4.662	2.29E−09	46	616 (8)

^a^ When appropriate, the induction of the corresponding gene is shown

Among the upstream regulators potentially involved in the observed transcriptional response, we found a significant overlap of the cytokines TNF, IL-1β, IL-6, MIF, and IFNβ1, as well as nitric oxide (Table 1; Fig. 1e). These responses are probably a secondary activation boost launched after the initial bacterial exposure. Interestingly, even though *Il10* expression was significantly increased upon *B. burgdorferi* stimulation (3.568 log_2_ fold induction, *p* = 1.42 × 10^−59^), the overall signature response significantly matched a repressed gene expression profile triggered by the IL-10 receptor (Table 1). These data support a proinflammatory response induced primarily by the interaction of *B. burgdorferi* with macrophages that is further amplified by secreted cytokines or the inhibition of anti-inflammatory pathways.

### The human peripheral blood monocyte transcriptional profile shows a similar global response but distinctive species-specific pattern upon *B. burgdorferi* stimulation

**Fig. 2:**
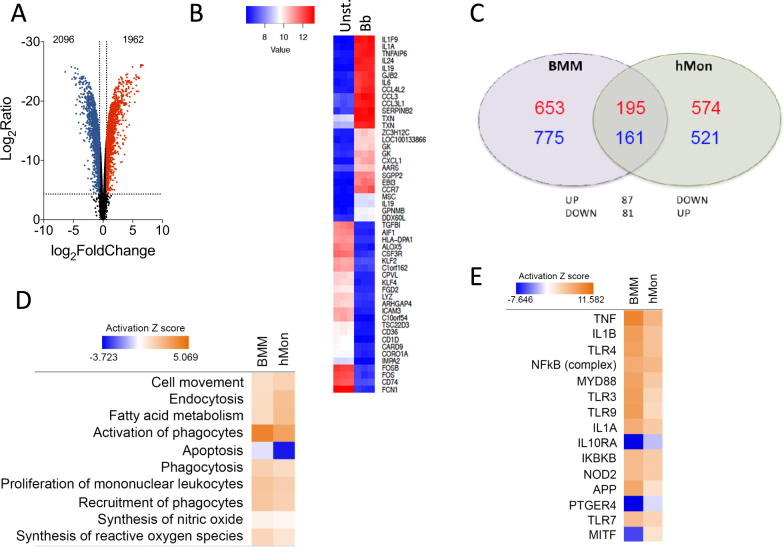
Analysis of genes regulated by *B. burgdorferi* in human CD14^+^ blood monocytes. **a** Volcano plot representing genes that are differentially regulated by stimulation with the spirochete. The red dots represent upregulated genes, whereas the blue dots correspond to those that are differentially downregulated. **b** Heat map showing the 50 most regulated genes in response to *B. burgdorferi* stimulation of human monocytes (Bb) compared with unstimulated monocytes (Unst.). **c** Venn diagram showing the overlap in gene regulation between BMMs and human monocytes stimulated with *B. burgdorferi*. The number of upregulated genes is represented in red. Downregulated genes are marked in blue. **d** Comparison of biological processes regulated in BMMs and hMon stimulated with *B. burgdorferi*. Processes that showed activation are indicated in orange, whereas those that were downregulated are presented in blue. The intensity of the colors is determined by the calculated *Z* value for each process. **e** Upstream activator pathways regulated by *B. burgdorferi* in BMMs and hMon. The colors and intensities are presented according to calculated *Z* values as in **d**

To corroborate the transcriptomic results obtained using murine BMMs and to test their biological relevance in the context of human innate immune responses to the spirochete, human CD14^+^ peripheral blood monocytes (hMon) were stimulated with *B. burgdorferi*. The transcriptional profile was obtained by microarray analysis and compared with BMM. Overall, 1962 genes were upregulated and 2096 downregulated (absolute value of log_2_ fold induction ≥ 1; *p* < 0.05; Fig. 2a), a selection of which were validated by qRT-PCR (Supplementary Figure S3) and represented clear patterns of expression (Fig. 2b). Reflecting the different cell types and origins (human vs. mouse), the transcriptional fingerprints showed a limited number of genes that were regulated and in the same direction in both cell types. Thus, 195 genes were upregulated both in BMMs and hMon upon stimulation with *B. burgdorferi*, whereas 161 genes were downregulated according to the established criteria (Fig. 2c). An additional 168 genes were regulated in opposite directions in both cells types (Fig. 2c). In spite of this, IPA showed a regulation of similar expression pathways, including cell movement and recruitment of leukocytes, fatty acid metabolism, endocytosis and phagocytosis, as well as the synthesis of nitric oxide and reactive oxygen species (Fig. 2d). Moreover, analysis of upstream regulators showed similar patterns of expression in response to pattern-recognition engagement (TLR3, TRL9, TLR7, NOD2), signaling intermediaries (MyD88, NF-κB complex, IKBKB) or secondary metabolites (TNF, IL-1β, IL-1α, IFNα2) (Fig. 2e). In both cases, the expression profile resembled the inhibition of gene expression initiated by IL-10Rα, whereas in a few cases the expression profile followed opposite directions in both cells types (i.e., MITF) (Fig. 2e). These results show that in both murine BMMs and hMon, stimulation with *B. burgdorferi* results in an overlapping proinflammatory profile that, nevertheless, reveals distinct transcriptional specificities.

### The proteome of BMMs shows a distinct profile upon stimulation of BMMs with *B. burgdorferi*

**Fig. 3:**
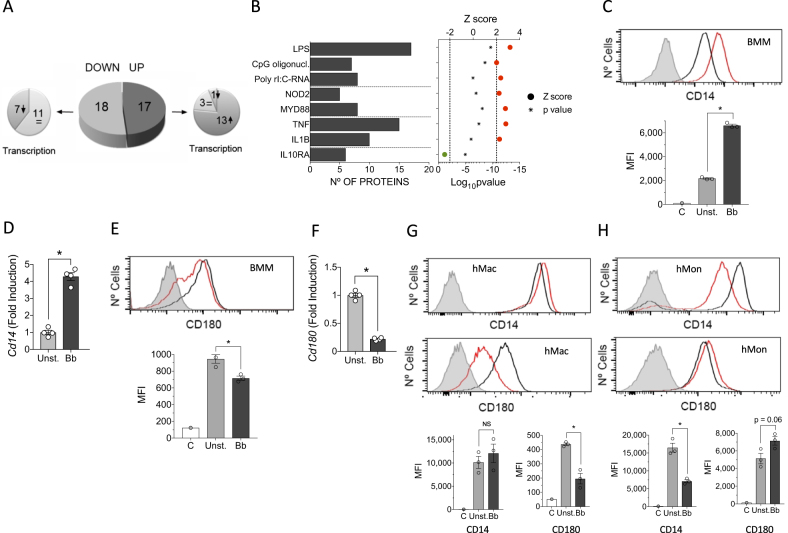
Proteomic analysis of BMMs stimulated with *B. burgdorferi*. **a** Pie chart showing the upregulated (dark gray) or downregulated proteins (light gray) in response to stimulation with the spirochete. The smaller pie charts on each side represent the direction of the regulation corresponding to the genes encoding the differentially expressed proteins. **b** Upstream regulators with significant *Z* scores (>2, red circles or <−2, green circle) and the number of regulated proteins. **c** Flow cytometry analysis of CD14 expression in BMMs stimulated with *B. burgdorferi* (red histogram) compared with unstimulated cells (black histogram). The gray histogram represents the unstained control. The average of the mean fluorescence intensity (MFI) ± SE for three independent mice is represented below. The experiment is representative of three performed. **d** Upregulation of *Cd14* expression levels determined by qRT-PCR of RNA extracted from *B. burgdorferi*-stimulated BMMs (black bar) or unstimulated controls (gray bar). The results represent the average ± SE of four independent mice and are representative of three experiments performed. **e** Flow cytometry analysis of CD180 expression in BMMs stimulated with *B. burgdorferi* (red histogram) compared with unstimulated cells (black histogram). The gray histogram represents the unstained control. The average of the MFI ± SE for three independent mice is shown below and is representative of three experiments. **f** Downregulation of *Cd180* expression levels by qRT-PCR in BMMs stimulated with *B. burgdorferi* (black bar) or unstimulated controls (gray bar). The results are the mean ± SE of 4 independent mice. **g** Expression of CD14 (top histograms) and CD180 (bottom histograms) in human monocyte-derived macrophages stimulated with *B. burgdorferi* (red histograms) and unstimulated controls (black histograms). The gray histogram represents the unstained controls. The average of the mean fluorescence intensity (MFI) ± SE for three independent determinations is represented below. The results are representative of those obtained with six independent samples. **h** Expression of CD14 (top histograms) and CD180 (bottom histograms) in hMon stimulated with *B. burgdorferi* (red histograms) and unstimulated controls (black histograms. The gray histogram represents the unstained controls. The average of the mean fluorescence intensity (MFI) ± SE for three independent determinations is represented below. The results are representative of those obtained with nine independent samples

We also assessed differences in protein levels between unstimulated and *B. burgdorferi*-stimulated BMMs using LF MS. We detected 810 proteins represented by at least 2 peptides, 35 of which showed significantly different levels (*p* < 0.05) and were regulated at least twofold (absolute value of log_2_ fold induction ≥ 1; 17 upregulated and 18 downregulated) between unstimulated and *B. burgdorferi*-stimulated BMMs (Fig. 3a; Supplementary Table S3). Of the 17 upregulated proteins, 13 corresponded to genes that were significantly upregulated upon stimulation in the transcriptomic approach, whereas 3 corresponded to genes with no significant transcriptional change and 1 was downregulated at the gene expression level (Fig. 3a; Table 2). In contrast, the majority of proteins that were downregulated upon stimulation with *B. burgdorferi* (11 of 18) corresponded to genes without changes in expression, and 7 also showed downregulation at the gene expression level (Fig. 3a; Table 2).

**Table 2:** Proteins significantly regulated by *B. burgdorferi* stimulation of BMMs and their corresponding gene transcription profiles

	

Proteins and genes that were regulated in the same direction are marked in green (downregulated) or orange (upregulated). IRG1 was regulated in opposite directions at the protein and gene expression levels (marked in gray)

Despite the limited amount of proteins that were significantly changed at this time interval of stimulation, IPA showed that the majority corresponded to proteins that are known to be regulated by LPS- (17 proteins), CpG oligonucleotide- (7 proteins) or poly rI:C-RNA-induced stimulation (8 proteins; Fig. 3b). Furthermore, several proteins corresponded to NOD2- (5 proteins) or MyD88-induced pathways (8 proteins). The regulated proteins were also related to the stimulation with TNF (15 proteins) or IL-1β (10 proteins) (Fig. 3b). As expected, the proteins that were regulated by IL-10Rα engagement (6 proteins) showed that this pathway is inhibited by *B. burgdorferi* stimulation (Fig. 3b). Overall, the proteomics analysis of BMMs corroborated the activation pathways initiated by stimulation with the spirochete at the gene expression level both in BMMs and hMon.

We then sought to identify proteins that are regulated by *B. burgdorferi* in macrophages and that may have a role in the response of these cells to the spirochete. Among the differentially expressed proteins, the majority represented plasma membrane-anchored proteins (12 proteins) (Table 3). Notably, we observed two proteins, CD14 and CD180, which were regulated in opposite directions. The stimulation of BMMs with *B. burgdorferi* resulted in increased levels of both surface CD14 and gene expression (Fig. 3c, d), whereas the levels of CD180, a non-canonical member of the TLR family of proteins, were significantly reduced (Fig. 3e, f). The downregulation of CD180 was corroborated in in vitro differentiated human macrophages (Fig. 3g). In contrast, human monocytes isolated from blood showed an opposite phenotype, with reduced levels of CD14 and increased expression of CD180 in response to the spirochete (Fig. 3h), evidencing the distinct role of both types of cells in tissue homeostasis and immunity^37^.

**Table 3:** Proteins significantly regulated by *B. burgdorferi* stimulation of BMMs that are located in the plasma membrane

**Protein**	**Genes**	**Name(s)**	**Function**
CTR2_MOUSE	*Slc7a2*	ATRC2	Cationic amino acid transporte
CLC4E_MOUSE	*Clec4e*	Mincle	C-type lectin
FCGR2_MOUSE	*Fcgr2b*	CD32, FC gamma RIIB	Low affinity IgG Fc receptor
ICAM1_MOUSE	*Icam1*	CD54, Intercellular adhesion molecule 1	Adhesion molecule
CD14_MOUSE	*Cd14*	CD14	Cooperates with TLR4 and CR3
4F2_MOUSE	*Slc3a2*	CD98, Ly10	Amino acid and Ca^2+^ Transporter
CD36_MOUSE	*Cd36*	CD36, Fatty acid translocase	Lipid binding
AP3B1_MOUSE	*Ap3b1*	Pearl, Tsap4	Protein targeting to lysosome
STOM_MOUSE	*Stom*	Stomatin	Regulation of ion channels and transporters
LRP1_MOUSE	*Lrp1*	CD91, alpha-2-macroglobulin receptor	Lipid homeostasis and clearance of apoptotic cells
CSF1R_MOUSE	*Csf1r*	CD115, CSF1 receptor	Regulation of macrophage function
CD180_MOUSE	*Cd180*	RP105, Ly78	Forms dimers with MD-1. Pattern-recognition receptor

### CD180 regulates the phagocytosis of *B. burgdorferi* and the production of TNF

**Fig. 4:**
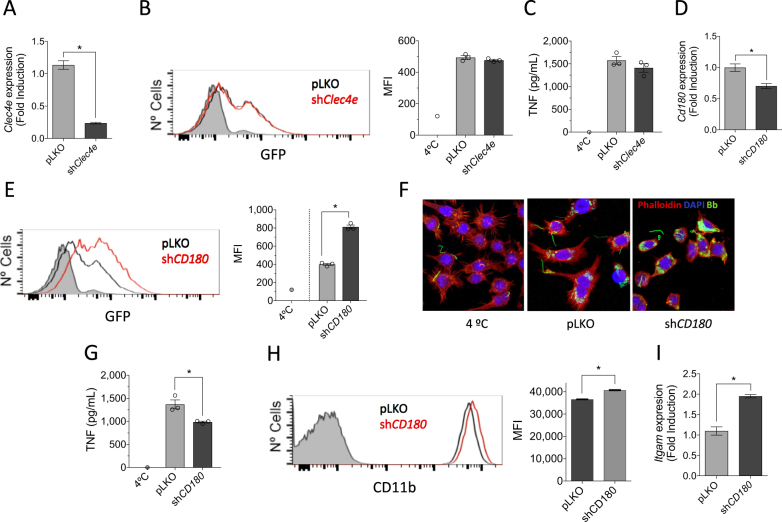
CD180 regulates the response of macrophages to *B. burgdorferi*. **a** Downregulation of *Clec4e* expression in RAW264.7 cells infected with a lentivirus containing specific shRNA (black bar) or an empty vector (pLKO, gray), as determined by qRT-PCR. The results represent the average of three determinations. **b** Phagocytosis of *B. burgdorferi* by RAW264.7 cells containing shRNA specific for *Clec4a* (red histogram) or an empty control (pLKO, black histogram). The gray histogram represents cells incubated at 4 °C (control). The average MFI of three determinations is presented on the right. The results are representative of at least three independent experiments. **c** TNF production upon stimulation with *B. burgdorferi* by RAW264.7 cells with silenced *Clec4a* or controls. **d** Silencing of *CD180* in RAW264.7 cells (black bar) or pLKO controls (gray bar), as determined by qRT-PCR. The values correspond to the average ± SE of three determinations and are representative of three experiments. **e**. Phagocytosis of *B. burgdorferi* by sh*Cd180* RAW264.7 cells (red histogram) or pLKO-infected controls (black histogram). The gray histogram represents the 4 °C control. The average of three determinations is presented on the right. The results are representative of at least three independent experiments. **f** Confocal micrograph of RAW264.7 cells containing shRNA specific for *Cd180* (shCD180) or an empty vector (pLKO). The cells were stained with phalloidin (red) and DAPI. *B. burgdorferi* are shown in green. **g** TNF production upon stimulation with *B*. burgdorferi by RAW264.7 cells containing a shRNA specific for *Cd180* or a vector control (pLKO). The average ± SE of three determinations is presented and represent at least three independent experiments. **h** Flow cytometry showing the expression of CD11b in unstimulated RAW264.7 cells containing a shRNA specific for *Cd180* (red histogram) or an empty vector (pLKO, black histogram). The gray histogram represents the unstained control. The average of the mean fluorescence intensity (MFI) ± SE for three determinations is represented on the right. **i** qRT-PCR showing the expression of the *Itgam* gene in unstimulated RAW264.7 cells containing an shRNA specific for *Cd180* (black bar) or the empty vector (pLKO, gray bar). The results represent the average ± SE of three determinations

Among the surface proteins regulated by the stimulation with *B. burgdorferi*, Clec4e (Mincle) has been shown to be involved in the phagocytosis of several microorganisms^38^. To address its potential role in the response of phagocytic cells to the spirochete, we silenced *Clec4e* (Mincle) in RAW264.7 cells by lentiviral infection (Fig. 4a). The repression of *Clec4e* gene expression did not result in an appreciable reduction of the phagocytic capacity of RAW264.7 cells (Fig. 4b). Furthermore, the analysis of TNF production upon stimulation with *B. burgdorferi* did not result in a differential production of this cytokine (Fig. 4c), indicating that the C-type lectin receptor is not involved in the internalization or proinflammatory cytokine production in response to the spirochete.

Phagocytosis of *B. burgdorferi* is largely dependent on MyD88-mediated signals^13,39^. However, the involved receptor(s) are still unknown. Since the stimulation with *B. burgdorferi* induced the downregulation of CD180 (Table 2), we sought to determine whether this TLR family member is involved in the internalization of the spirochete and/or the induction of proinflammatory responses. Silencing of *Cd180* (Fig. 4d) resulted in a significant increase in the capacity of RAW264.7 cells to internalize *B. burgdorferi* (Fig. 4e, f). Strikingly, the repression of *Cd180* also resulted in a significant reduction in TNF production by RAW264.7 cells in response to the spirochete (Fig. 4g). The stimulation of Cd180-silenced cells also resulted in lower mRNA levels of *Tnf* and *Il6* (Supplementary Figure S4). Phagocytosis of *B. burgdorferi* is mediated by several, largely unknown, phagocytic receptors, including complement receptor (CR) 3 (CD11b/CD18)^13^. Interestingly, phagocytosis mediated by CR3 results in the attenuation of the inflammatory response, particularly the production of TNF^13^. In concordance with our results, CD180 has been associated with the tempering of the proinflammatory response of macrophages^40,41^. We sought to determine whether the increased phagocytosis of *B. burgdorferi* in cells with repressed expression of *Cd180* was due to the regulation of CR3. The analysis of CD11b surface expression by flow cytometry showed significantly increased levels in sh*Cd180* cells compared with the controls (Fig. 4h). Furthermore, the analysis by qRT-PCR of sh*Cd180* cells showed the upregulation of *Itgam* gene expression (Fig. 4i).

Overall, these data unravel a new role for CD180 in the regulation of expression of the phagocytic receptor for *B. burgdorferi*, CR3. The inhibition of CR3 expression by CD180 attenuates phagocytosis and promotes proinflammatory responses. These results define CD180 as an important regulator of immune responses mediated by *B. burgdorferi*.

## Discussion

Macrophages can recognize, phagocytose, and eliminate invading pathogens and thus have a crucial role in host defense. These cells constitute a key component of the innate immune system with the ability to respond to a wide array of stimuli, both endogenous and pathogen-associated, showing an enormous capacity to adapt and respond to environmental cues. In the presence of pathogens, macrophages produce an array of inflammatory factors upon the engagement of pattern-recognition receptors (PRRs). The response of macrophages is further modulated by environmental signals, including cytokines such as TNF, IL-10, IL-6, or interferons, among other factors. The use of high-throughput (omic) techniques has grown exponentially in recent years driven by marked improvement in analytical platforms, increasing resolution and sensitivity, high-throughput capabilities and reducing costs^42^. These methodologies have allowed the elucidation of mechanisms of pathogenesis for disease-causing agents, identified disease biomarkers (biosignatures) or characterized the response to preventative and therapeutic interventions^42,43^. Omic technologies have been used in Lyme borreliosis patients, enabling the determination of a distinctive disease biosignature, particularly at the early disease stages^44–46^. Here we employed high-throughput techniques to study biosignatures of both primary murine macrophages and human monocytes exposed to *B. burgdorferi*. Our results showed that although general transcriptional traits are shared between human monocytes and murine macrophages, there are several important differences between both cell types. However, both their expression profiles resemble the transcriptional fingerprint upon recognition of bacteria and viruses by PRRs, including TLR signaling or the activation of IRF, evidencing the complex nature of the interaction signature between *B. burgdorferi* and phagocytic cells, which depends on several receptors. Moreover, comparison of the signaling pathways activated by *B. burgdorferi* on isolated murine macrophages and human peripheral blood monocytes resemble those found in peripheral blood monocytic cells obtained from patients diagnosed with Lyme borreliosis^44^ (Supplementary Figure S5) and correlates with a previous description of the transcriptome induced by *B. burgdorferi* stimulation of the murine cell line J774^47^ (Supplementary Figure S6). Overall, these data suggest that the identification of transcriptional traits in isolated cells stimulated with the spirochete can represent biosignatures that are present in infected individuals.

Inherent to their killing capacity, macrophages produce numerous molecules that, while exerting functions related to host defenses, are also capable of damaging host tissue^48^. Inflammation is, therefore, a two-edged sword and, thus, coupling of phagocytosis to inflammation must be tightly regulated. It is not surprising that many regulatory mechanisms are required to control the inflammatory response by preventing inappropriate activation or by a timely termination of the immune response. *B. burgdorferi* stimulation of TLR-dependent and independent signaling in host cells leads to transcriptional activation, the release of inflammatory mediators, and anti-microbial responses^49–52^. The stimulation with *B. burgdorferi* also leads to the induction of several anti-inflammatory pathways, including IL-10^53–56^, PPAR^34–36^, or the induction of phagocytic receptors with anti-inflammatory action, such as CR3^13,57^. The relative contribution of each is unknown, but our results show that even though IL-10 production is highly induced by the spirochete, IL-10R-dependent signaling is repressed at the time of analysis. However, our data show that the stimulation of macrophages with *B. burgdorferi* induces the downregulation of CD180. Furthermore, this stimulation also results in decreased expression of the *Ly86* gene (encoding the CD180 accessory molecule MD-1; −1.902 log_2_ fold induction, *p* = 1.09 × 10^−15^ in *B. burgdorferi*-stimulated BMMs compared with unstimulated cells), which is required for the function and surface expression of CD180^58^. In turn, the repression of CD180 expression results in the secondary upregulation of CR3 and the tempering of the inflammatory response. Overall, these results suggest that the initial stimulation with the spirochete sets the starting point for the control of the proinflammatory output of macrophages, which may be more evident further downstream in the activation process. Whether the sustained exposure of phagocytic cells with *B. burgdorferi* reflects the increased activation of these anti-inflammatory pathways will be the subject of further investigation.

Our results unravel a mechanism involving CD180 to control the response of macrophages to *B. burgdorferi*. CD180 (also known as RP105) is a TLR-like protein that is expressed by B-lymphocytes, macrophages and dendritic cells^59^. CD180 is unique in its role in both enhancing and suppressing TLR responses that seem to vary with the cell type^59^. CD180 is required for full responsiveness to LPS in B cells^58,60^, while it seems to differentially regulate TLR4- and TLR2-induced responses in dendritic cells and macrophages^59^. The response to *B. burgdorferi* is more complex than the surface interaction between PAMPs and PRRs and is dependent on the internalization of the spirochete^12^. In turn, different phagocytosis pathways lead to proinflammatory responses or the downregulation of the production of cytokines such as TNF^13^. Silencing *Cd180* increased *B. burgdorferi* phagocytosis by macrophages while reducing TNF synthesis, suggesting that the TLR molecule modulates the expression of phagocytic receptors. CR3 is a phagocytic receptor that recognizes and phagocytose the spirochete, leading to a decrease in TNF production^13^. Similarly, engagement of CR3 during phagocytosis of apoptotic cells downregulates the production and secretion of proinflammatory cytokines^61^. Now, we describe a novel functional interaction between CD180 and CR3 upon *B. burgdorferi* recognition. *B. burgdorferi* is able to modulate CD180 expression, which further regulates CR3 expression and subsequent CR3-mediated phagocytosis and cytokine production, providing an additional mechanism for the regulation of the immune responses triggered by *B. burgdorferi*. This mechanism of inflammation modulation has the advantage of providing an increase phagocytic activity to macrophages (that is required while the presence of spirochetes is still high), whereas at the same time tempering the induced proinflammatory response.

Overall, our data provide a global view of the intricate response mechanisms associated with the interaction of monocytes/macrophages with *B. burgdorferi*. These data also offer clues to mechanisms of control of the phagocytic and proinflammatory activity of these cells and suggest multiple mechanisms of control that may be relevant at different phases of the response.

## Electronic supplementary material

Supplemental Materials
