# Supplementary material for: A multi-omic analysis reveals the regulatory role of CD180 during the response of macrophages to Borrelia burgdorferi
Source: Emerg Microbes Infect. 2018 Mar 7;7:19. doi: 10.1038/s41426-017-0018-5 (PMC5841238; doi:10.1038/s41426-017-0018-5)
Supplement: Supplementary file 1 — Supplemental Materials [file 41426_2017_18_MOESM1_ESM.docx]

**A multi-omic analysis reveals the regulatory role of CD180 during the response of macrophages to *Borrelia burgdorferi***

Ana Carreras-González ^1^, Nicolás Navasa ^1^, Itziar Martin-Ruiz ^1^, José Luis Lavín ^2^, Mikel Azkargorta ^3,4^, Estíbaliz Atondo ^1^, Diego Barriales ^1^, Nuria Macías-Cámara ^2^, Miguel Angel Pascual-Itoiz ^1^, Leticia Sampedro ^1^, Julen Tomás-Cortázar ^1^, Ainize Peña-Cearra ^1,5^, Aize Pellón ^1^, Rafael Prados-Rosales ^1^, Leticia Abecia ^1^, Félix Elortza ^3,4^, Ana M. Aransay ^2,6^, Héctor Rodríguez ^1^ and Juan Anguita ^1,7,*^

Macrophage and Tick Vaccine Laboratory ^1^, Genome Analysis Platform ^2^ and Proteomics Platform ^3^, CIC bioGUNE, 48160 Derio, Bizkaia, Spain.

^4^ Carlos III Networked Proteomics Platform (ProteoRed-ISCIII), Universidad Autónoma de Madrid, 28049 Cantoblanco, Madrid, Spain.

^5^ Universidad del País Vasco/Euskal Herriko Unibertsitatea, 48940 Leioa, Bizkaia, Spain.

^6^ Centro de Investigación Biomédica en Red de enfermedades hepáticas y digestivas (CIBERehd), Instituto de Salud Carlos III, 28029 Madrid, Spain.

^7^ Ikerbasque, Basque Foundation for Science. 48011 Bilbao, Bizkaia, Spain.

Running Title: Multi-omic analysis of macrophages to *Borrelia*

Email Addresses: ACG: [acarreras@cicbiogune.es](mailto:acarreras@cicbiogune.es); NN: [nnavasa@cicbiogune.es](mailto:nnavasa@cicbiogune.es); IMR: [imartin@cicbiogune.es](mailto:imartin@cicbiogune.es); JLL: [jllavin@cicbiogune.es](mailto:jllavin@cicbiogune.es); MA: [mazkargorta@cicbiogune.es](mailto:mazkargorta@cicbiogune.es); EA: [eatondo@cicbiogune.es](mailto:eatondo@cicbiogune.es); DB: [dbarriales@cicbiogune.es](mailto:dbarriales@cicbiogune.es); NMC: [nmacias@cicbiogune.es](mailto:nmacias@cicbiogune.es); MAPI: [mpascual@cicbiogune.es](mailto:mpascual@cicbiogune.es); LS: [lsampedro@cicbiogune.es](mailto:lsampedro@cicbiogune.es); JTC: [jcortazar@cicbiogune.es](mailto:jcortazar@cicbiogune.es); APC: [apcearra@cicbiogune.es](mailto:apcearra@cicbiogune.es); AP; [apellon@cicbiogune.es](mailto:apellon@cicbiogune.es); RPR: [rprados@cicbiogune.es](mailto:rprados@cicbiogune.es); LA: [labecia@cicbiogune.es](mailto:labecia@cicbiogune.es); FE: [felortza@cicbiogune.es](mailto:felortza@cicbiogune.es); AMA: [amaransay@cicbiogune.es](mailto:amaransay@cicbiogune.es); HR: [hrodriguez@cicbiogune.es](mailto:hrodriguez@cicbiogune.es); JA: [janguita@cicbiogune.es](mailto:janguita@cicbiogune.es)

^*^ Correspondence: Juan Anguita, PhD. CIC bioGUNE. Parque Tecnológico de Bizkaia, 48160 Derio, Bizkaia, Spain. +34 944 061 311. [janguita@cicbiogune.es](mailto:janguita@cicbiogune.es)

**Supplementary Figure S1 Sample distance matrix of BMMs stimulated with *B. burgdorferi* (Bb) and unstimulated controls (U)**

**Supplementary Figure S2 Validation of the RNAseq on selected genes**

Fold induction changes by qRT-PCR of a group of selected genes differentially regulated in BMMs from 4 independent mice stimulated with *B. burgdorferi* (black bars) compared to unstimulated controls (grey bars). (A) Genes upregulated. (B) Genes downregulated.

**Supplementary Figure S3 Validation of the μArray on selected genes**

Fold induction changes by qRT-PCR of a group of selected genes differentially regulated in human monocytes from 3 independent donors stimulated with *B. burgdorferi* (black bars) compared to unstimulated controls (grey bars). (A) Genes upregulated. (B) Genes downregulated.

**Supplementary Figure S4 *Tnf* and *Il6* gene expression induced by *B. burgdorferi* in RAW264.7 cells with silenced *Cd180***

Control- and *Cd180*-silenced cells (10^6^ cells /ml) were stimulated with *B. burgdorferi* at an m.o.i. of 25 for 4h. Total RNA was then extracted, reverse-transcribed and used to determine by qRT-PCR for the levels of *Tnf* and *Il6* mRNA levels. The results represent the mean ± SE of triplicates.

**Supplementary Figure S5 Comparison of the signaling pathways induced by B. burgdorferi in murine BMMs, human monocytes and PBMCs from Lyme borreliosis patients**

Pathways identified by IPA induced by the stimulation with *B. burgdorferi* or human peripheral blood monocytes (A) or murine BMMs (B) compared to those identified in PBMCs isolated from diagnosed Lyme borreliosis patients (1). The colors represent the calculated z values and are indicated as activated (orange) or repressed (blue). Pathways marked in white were not identified by IPA as regulated.

A

B

**Supplementary Figure S6 Comparison between the transcriptome of the murine cell line J774 and BMM stimulated with *B. burgdorferi***

Correlation between the transcriptome of the murine macrophage cell line, J774 stimulated with *B. burgdorferi* for 24h (2) and BMMs stimulated with the spirochete for 16h. The data corresponding to the J774 cells correspond to a uArray analysis and extracted from the supplementary materials of the study. The correlation was calculated for those genes in which a change in transcriptional levels between stimulated and unstimulated cells was found, and correspond to those with an absolute value for the log_2_FoldInduction >= 1 and p < 0.05. The genes most regulated are identified.

| **Supplementary Table S1 Primers used** | | | | | |
| --- | --- | --- | --- | --- | --- |
|  |  |  |  |  |  |
| **Gene (m = mouse/ h = human)** | **Forward** | **Reverse** | **Tm** | **Purpose** | |
|  |  |  |  |  |  |
| *Rpl19* (m) | GACCAAGGAAGCACGAAAGC | CAGGCCGCTATGTACAGACA | 60.0 | Reference, RNAseq validation | |
| *Clec4e* (m) | CAACCACACATTTGACTTCTCTT | TCAGTGAGGTGGATAGGAGCA | 59/61 | RNAseq validation | |
| *Cd14* (m) | ATAGGAACCCTAGCCCAGATGA | ACAAGGCCACTGCTTGGGAT | 62.2/60.0 | RNAseq validation | |
| *Clec4d* (m) | GGAAAGTCATTCCAGACCCA | AAGACGCCATTTAACCCACA | 58.0/56.0 | RNAseq validation | |
| C*d180* (m) | CTTGACTGCACTTGCTCAAACA | CCCACAGCTGCCATACTACA | 60.0 | RNAseq validation | |
| *Siglec5* (m) | TGTGCGTCTTTGTAGCCTGC | *CACTGGAGAGCCGCTGAAT* | 60.0/59.0 | RNaseq validation | |
| *Syk* (m) | GATGGGCTCTACCTGCTACG | CCATCAGGTTCCTGGGAGTG | 63.0 | RNAseq validation | |
| *Ptpre* (m) | ACCAAGCCTTACTGGAATACTACC | CTGTGCTATGGAGCGTCTGC | 64.0/63.0 | RNAseq validation | |
| *Ly86* (m) | TGGAACATCAAGTCCAGGGT | TCCTATCCCCTTTGTGAGGA | 58.0 | RNAseq validation | |
| *Clec10a* (m) | AACCTTCCGCTGGATCTGTG | GTTGAGACCGGGTAGGAGGA | 60.0/63 | RNAseq validation | |
| *RANBP1 (h)* | CATGACCCTCAGTTTGAGCCA | CTCGCTCCTTCCATTCTGGG | 60.2 | Reference, mArray | |
| *PLXNC1 (h)* | GGGAGCAAAATGGCTGCTTG | ACAGAAGGGTTCCCACGGTA | 60.4 | Reference, mArray | |
| *CD40 (h)* | AGGAGATCAATTTTCCCGACGA | CTCACTGTCTCTCCTGCACT | 59.5/58.7 | mArray validation | |
| *IL1A (h)* | AGGCATCCTCCACAATAGCAG | GGACCAATTACTGGCTCAAGGT | 59.8/60.2 | mArray validation | |
| *IL6 (h)* | GTGGCTGCAGGACATGACAA | GCCCAGTGGACAGGTTTCTG | 60.8 | mArray validation | |
| *FCGR1A (h)* | CTCCTTCTACATGGGCAGCAA | GGCTGCGCTTAAGGACATTT | 60/59.1 | mArray validation | |
| *ITGA5 (h)* | GCAGAATTTGGGTTCTGCCT | GGGCACTGGGTCAGATTTATTG | 58.7/59.3 | mArray validation | |
| *FCGR2A (h)* | CTTGGGGAAGACGAAGGGATG | CACAGAAGGTGCAGTCAGTCA | 60.4/60.2 | mArray validation | |
| *FCGRT (h)* | AGGCCCAGGATGCTGATTTG | AACTAGGCCCAGAACCCCTT | 60.4 | mArray validation | |
| *IL10 (h)* | GCTGTTGAGCTGTTTTCCCTG | TCCGAGACACTGGAAGGTGA | 60/60.2 | mArray validation | |
| *LY86 (h)* | AAAGAAGCGCCCCCAAAGAG | GGGAGGCGACCAATTAGAGA | 60.9/ 59 | mArray validation | |
|  |  |  |  |  |  |
| *Itgam (m)* | TGTACCACTCATTGTGGGCA | GTCCATCAGCTTCGGTGTTG | 58/60 | qRT-PCR |  |

| **Supplementary Table S2** **Genes associated with phagocytosis and activation of phagocytes and their corresponding regulation in both BMMs and hMon stimulated with *B. burgdorferi* compared to unstimulated samples**  Values in bold represent significantly regulated genes. Those genes regulated in the same direction are marked either in orange (upregulated in both analysis) or green (downregulated). | | | | | | | | | | | |
| --- | --- | --- | --- | --- | --- | --- | --- | --- | --- | --- | --- |
| **Mouse** | **Human** | **Entrez Gene Name** | **BMM** | | | | **hMon** | | | |  |
|  |  |  | **Log2(Ratio)** | | **p-value** | | **Log2(Ratio)** | | **p-value** | |  |
| Phagocytosis | | | | | | | | | | |  |
| Cd38 | CD38 | CD38 molecule | **9.924** | | **3.33E-50** | | 0.013 | | 9.13E-01 | |  |
| sf3 | CSF3 | colony stimulating factor 3 | **9.086** | | **1.58E-52** | | **1.385** | | **3.75E-05** | |  |
| Il1b | IL1B | interleukin 1 beta | **7.704** | | **7.83E-23** | | **2.474** | | **7.42E-07** | |  |
| Il6 | IL6 | interleukin 6 | **7.634** | | **2.72E-22** | | **6.014** | | **6.61E-08** | |  |
| Saa1 | SAA1 | serum amyloid A1 | **6.811** | | **2.75E-16** | | NA | | NA | |  |
| Syt7 | SYT7 | synaptotagmin 7 | **6.429** | | **5.80E-18** | | -0.069 | | 5.17E-01 | |  |
| Cav1 | CAV1 | caveolin 1 | **6.385** | | **8.93E-69** | | **1.451** | | **3.80E-04** | |  |
| Adora2a | ADORA2A | adenosine A2a receptor | **6.002** | | **5.85E-70** | | **2.929** | | **3.23E-07** | |  |
| Adora2b | ADORA2B | adenosine A2b receptor | **5.632** | | **9.01E-136** | | -0.391 | | 1.40E-02 | |  |
| Lcn2 | LCN2 | lipocalin 2 | **5.578** | | **4.55E-11** | | -0.077 | | 5.47E-01 | |  |
| Tnf | TNF | tumor necrosis factor | **5.2** | | **5.17E-77** | | **3.336** | | **7.92E-05** | |  |
| Clec4n | CLEC6A | C-type lectin domain family 6 member A | **4.759** | | **7.15E-33** | | 0.035 | | 8.01E-01 | |  |
| Isg15 | ISG15 | ISG15 ubiquitin-like modifier | **4.731** | | **2.97E-34** | | **-1.692** | | **1.93E-02** | |  |
| Spic | SPIC | Spi-C transcription factor | **4.584** | | **1.06E-23** | | -0.078 | | 3.98E-01 | |  |
| F10 | F10 | coagulation factor X | **4.091** | | **7.04E-07** | | NA | | NA | |  |
| Mir125a | mir-10 | microRNA 100 | **4.054** | | **3.78E-04** | | -0.053 | | 7.18E-01 | |  |
| Src | SRC | SRC proto-oncogene. non-receptor tyrosine kinase | **3.85** | | **2.93E-22** | | **2.341** | | **2.13E-07** | |  |
| Hmox1 | HMOX1 | heme oxygenase 1 | **3.722** | | **3.37E-93** | | **-1.424** | | **4.92E-06** | |  |
| Fpr1 | FPR1 | formyl peptide receptor 1 | **3.666** | | **1.18E-03** | | **-1.858** | | **1.12E-06** | |  |
| Mirlet7e | let-7 | microRNA let-7a-1 | **3.642** | | **2.10E-03** | | 0.145 | | 1.06E-01 | |  |
| Il10 | IL10 | interleukin 10 | **3.568** | | **1.42E-59** | | **1.929** | | **3.52E-05** | |  |
| Ehd1 | EHD1 | EH domain containing 1 | **3.535** | | **1.02E-29** | | **1.261** | | **4.08E-05** | |  |
| Cxcl10 | CXCL10 | C-X-C motif chemokine ligand 10 | **3.487** | | **1.09E-04** | | NA | | NA | |  |
| Nod2 | NOD2 | nucleotide binding oligomerization domain containing 2 | **3.373** | | **5.60E-62** | | **-2.011** | | **4.88E-05** | |  |
| Pla2g4a | PLA2G4A | phospholipase A2 group IVA | **3.261** | | **4.82E-52** | | NA | | NA | |  |
| Fas | FAS | Fas cell surface death receptor | **3.204** | | **6.59E-05** | | NA | | NA | |  |
| Irf7 | IRF7 | interferon regulatory factor 7 | **3.163** | | **5.20E-20** | | -0.353 | | 2.17E-01 | |  |
| Csf2 | CSF2 | colony stimulating factor 2 | **3.14** | | **1.16E-02** | | **3.814** | | **9.01E-06** | |  |
| Pf4 | PF4 | platelet factor 4 | **3.13** | | **2.76E-48** | | NA | | NA | |  |
| Ly75 | LY75 | lymphocyte antigen 75 | **2.954** | | **9.11E-06** | | NA | | NA | |  |
| Cebpb | CEBPB | CCAAT/enhancer binding protein beta | **2.768** | | **3.05E-28** | | 0.332 | | 5.43E-03 | |  |
| Fcgr2b | FCGR2B | Fc fragment of IgG receptor IIb | **2.708** | | **2.48E-23** | | 0.621 | | 1.19E-01 | |  |
| Camp | CAMP | cathelicidin antimicrobial peptide | **2.677** | | **8.79E-03** | | -0.293 | | 8.35E-03 | |  |
| Pla2g5 | PLA2G5 | phospholipase A2 group V | **2.588** | | **7.69E-03** | | NA | | NA | |  |
| Ets2 | ETS2 | ETS proto-oncogene 2. transcription factor | **2.475** | | **4.73E-94** | | **1.896** | | **1.80E-05** | |  |
| Tlr1 | TLR1 | toll like receptor 1 | **2.446** | | **3.21E-11** | | NA | | NA | |  |
| Msr1 | MSR1 | macrophage scavenger receptor 1 | **2.437** | | **3.36E-59** | | NA | | NA | |  |
| Cd14 | CD14 | CD14 molecule | **2.32** | | **7.36E-21** | | -0.236 | | 7.25E-01 | |  |
| Slc11a1 | SLC11A1 | solute carrier family 11 member 1 | **2.316** | | **1.79E-34** | | **-1.139** | | **1.24E-03** | |  |
| Plaur | PLAUR | plasminogen activator. urokinase receptor | **2.309** | | **1.07E-45** | | **1.326** | | **5.17E-05** | |  |
| Ccl2 | Ccl2 | chemokine (C-C motif) ligand 2 | **2.255** | | **5.73E-12** | | NA | | NA | |  |
| Cd302 | CD302 | CD302 molecule | **2.16** | | **1.36E-08** | | **-1.94** | | **1.36E-04** | |  |
| Icam1 | ICAM1 | intercellular adhesion molecule 1 | **2.138** | | **1.48E-03** | | **1.319** | | **5.39E-05** | |  |
| Abca1 | ABCA1 | ATP binding cassette subfamily A member 1 | **1.953** | | **3.56E-14** | | **-1.215** | | **2.49E-02** | |  |
| Cybb | CYBB | cytochrome b-245 beta chain | **1.765** | | **3.11E-07** | | 0.19 | | 2.85E-01 | |  |
| Mapkapk2 | MAPKAPK2 | mitogen-activated protein kinase-activated protein kinase 2 | **1.763** | | **7.23E-16** | | -0.258 | | 1.41E-02 | |  |
| Eif2ak2 | EIF2AK2 | eukaryotic translation initiation factor 2 alpha kinase 2 | **1.758** | | **9.08E-23** | | **-1.448** | | **1.05E-03** | |  |
| Csf2rb | CSF2RB | colony stimulating factor 2 receptor beta common subunit | **1.653** | | **1.08E-28** | | 0.074 | | 4.47E-01 | |  |
| Thbs1 | THBS1 | thrombospondin 1 | **1.613** | | **8.43E-23** | | 0.292 | | 7.83E-01 | |  |
| Hck | HCK | HCK proto-oncogene. Src family tyrosine kinase | **1.561** | | **3.80E-19** | | **1.842** | | **1.87E-05** | |  |
| Tlr2 | TLR2 | toll like receptor 2 | **1.556** | | **1.25E-02** | | 0.316 | | 1.11E-02 | |  |
| Syk | SYK | spleen associated tyrosine kinase | **1.491** | | **1.74E-22** | | -0.2 | | 1.06E-01 | |  |
| Ptx3 | PTX3 | pentraxin 3 | **1.457** | | **4.10E-02** | | NA | | NA | |  |
| Scarf1 | SCARF1 | scavenger receptor class F member 1 | **1.452** | | **5.74E-07** | | 0.562 | | 1.65E-03 | |  |
| Prkce | PRKCE | protein kinase C epsilon | **1.378** | | **1.14E-04** | | NA | | NA | |  |
| Mif | MIF | macrophage migration inhibitory factor (glycosylation-inhibiting factor) | **1.367** | | **3.53E-23** | | -0.241 | | 2.29E-01 | |  |
| Pip5k1a | PIP5K1A | phosphatidylinositol-4-phosphate 5-kinase type 1 alpha | **1.234** | | **1.46E-14** | | 0.013 | | 9.21E-01 | |  |
| Cat | CAT | catalase | **1.223** | | **2.50E-08** | | **-1.656** | | **7.41E-06** | |  |
| Treml2 | TREML2 | triggering receptor expressed on myeloid cells like 2 | **1.196** | | **3.56E-03** | | NA | | NA | |  |
| Abca7 | ABCA7 | ATP binding cassette subfamily A member 7 | **1.183** | | **1.42E-04** | | -0.933 | | 4.90E-04 | |  |
| Serpine1 | SERPINE1 | serpin family E member 1 | **1.126** | | **1.20E-02** | | 0.075 | | 4.50E-01 | |  |
| Myh9 | MYH9 | myosin heavy chain 9 | **1.104** | | **3.56E-21** | | -0.25 | | 2.47E-02 | |  |
| Ptger2 | PTGER2 | prostaglandin E receptor 2 | **1.093** | | **1.01E-04** | | 0.668 | | 2.48E-03 | |  |
| Coro1a | CORO1A | coronin 1A | **-1.034** | | **2.36E-05** | | **-2.956** | | **8.71E-08** | |  |
| Itgax | ITGAX | integrin subunit alpha X | **-1.042** | | **3.89E-05** | | 0.612 | | 1.19E-02 | |  |
| Swap70 | SWAP70 | SWAP switching B-cell complex subunit 70 | **-1.062** | | **4.54E-15** | | 0.548 | | 3.66E-03 | |  |
| Rab31 | RAB31 | RAB31. member RAS oncogene family | **-1.064** | | **3.56E-13** | | **1.144** | | **4.86E-04** | |  |
| Padi4 | PADI4 | peptidyl arginine deiminase 4 | **-1.096** | | **4.00E-02** | | **-3.58** | | **1.82E-06** | |  |
| Mfge8 | MFGE8 | milk fat globule-EGF factor 8 protein | **-1.117** | | **2.43E-06** | | -0.324 | | 7.07E-02 | |  |
| Cerk | CERK | ceramide kinase | **-1.159** | | **5.82E-16** | | -0.401 | | 2.35E-02 | |  |
| Vwf | VWF | von Willebrand factor | **-1.173** | | **1.75E-09** | | -0.323 | | 8.45E-03 | |  |
| P2rx7 | P2RX7 | purinergic receptor P2X 7 | **-1.208** | | **8.56E-06** | | **2.948** | | **5.35E-05** | |  |
| Lgals3 | LGALS3 | galectin 3 | **-1.215** | | **7.76E-10** | | 0.936 | | 8.72E-04 | |  |
| Fgr | FGR | FGR proto-oncogene. Src family tyrosine kinase | **-1.23** | | **3.64E-02** | | -0.35 | | 1.64E-02 | |  |
| Ptger4 | PTGER4 | prostaglandin E receptor 4 | **-1.266** | | **2.12E-04** | | 0.485 | | 1.76E-01 | |  |
| Camk1d | CAMK1D | calcium/calmodulin dependent protein kinase ID | **-1.28** | | **5.95E-10** | | -0.139 | | 1.58E-01 | |  |
| Dock1 | DOCK1 | dedicator of cytokinesis 1 | **-1.282** | | **3.87E-06** | | NA | | NA | |  |
| Myc | MYC | v-myc avian myelocytomatosis viral oncogene homolog | **-1.288** | | **9.08E-04** | | -0.578 | | 7.59E-03 | |  |
| Rora | RORA | RAR related orphan receptor A | **-1.292** | | **1.26E-05** | | NA | | NA | |  |
| Mkl1 | MKL1 | megakaryoblastic leukemia (translocation) 1 | **-1.326** | | **1.58E-13** | | -0.285 | | 7.14E-03 | |  |
| Rras2 | RRAS2 | related RAS viral (r-ras) oncogene homolog 2 | **-1.386** | | **8.46E-03** | | NA | | NA | |  |
| Gsn | GSN | gelsolin | **-1.645** | | **2.84E-13** | | -0.676 | | 2.91E-03 | |  |
| Elmo1 | ELMO1 | engulfment and cell motility 1 | **-1.783** | | **3.43E-10** | | -0.868 | | 1.36E-04 | |  |
| Gp1ba | GP1BA | glycoprotein Ib platelet alpha subunit | **-1.814** | | **5.75E-03** | | -0.07 | | 5.32E-01 | |  |
| Tecpr1 | TECPR1 | tectonin beta-propeller repeat containing 1 | **-1.888** | | **1.75E-32** | | -0.372 | | 5.49E-02 | |  |
| Trem2 | TREM2 | triggering receptor expressed on myeloid cells 2 | **-2.026** | | **7.70E-21** | | NA | | NA | |  |
| Fyn | FYN | FYN proto-oncogene. Src family tyrosine kinase | **-2.069** | | **8.63E-14** | | **-1.045** | | **6.38E-05** | |  |
| Mcoln3 | MCOLN3 | mucolipin 3 | **-2.07** | | **1.08E-14** | | 0.561 | | 2.10E-02 | |  |
| Colec12 | COLEC12 | collectin subfamily member 12 | **-2.108** | | **2.00E-17** | | NA | | NA | |  |
| Plau | PLAU | plasminogen activator. urokinase | **-2.119** | | **9.07E-10** | | 0.574 | | 3.43E-03 | |  |
| Ucp2 | UCP2 | uncoupling protein 2 | **-2.129** | | **5.52E-24** | | **-1.085** | | **1.34E-04** | |  |
| Pros1 | PROS1 | protein S (alpha) | **-2.172** | | **4.41E-23** | | -0.022 | | 8.73E-01 | |  |
| Osbp2 | OSBP2 | oxysterol binding protein 2 | **-2.175** | | **1.77E-03** | | NA | | NA | |  |
| Rapgef3 | RAPGEF3 | Rap guanine nucleotide exchange factor 3 | **-2.194** | | **1.30E-08** | | NA | | NA | |  |
| Megf10 | MEGF10 | multiple EGF like domains 10 | **-2.239** | | **2.74E-02** | | NA | | NA | |  |
| C1qa | C1QA | complement C1q A chain | **-2.259** | | **1.05E-41** | | NA | | NA | |  |
| Klf2 | KLF2 | Kruppel like factor 2 | **-2.301** | | **3.51E-10** | | **-3.7** | | **7.14E-08** | |  |
| Pld4 | PLD4 | phospholipase D family member 4 | **-2.302** | | **9.52E-12** | | 0.049 | | 6.26E-01 | |  |
| Mex3b | MEX3B | mex-3 RNA binding family member B | **-2.38** | | **1.29E-07** | | -0.166 | | 1.43E-01 | |  |
| Anxa1 | ANXA1 | annexin A1 | **-2.447** | | **1.40E-51** | | -0.468 | | 6.67E-02 | |  |
| Hgf | HGF | hepatocyte growth factor | **-2.566** | | **5.64E-17** | | NA | | NA | |  |
| Rsph1 | RSPH1 | radial spoke head 1 homolog | **-2.611** | | **1.03E-02** | | NA | | NA | |  |
| Clec7a | CLEC7A | C-type lectin domain family 7 member A | **-2.776** | | **1.84E-38** | | -0.503 | | 4.31E-04 | |  |
| Fgf10 | FGF10 | fibroblast growth factor 10 | **-2.785** | | **2.85E-02** | | NA | | NA | |  |
| Rab27a | RAB27A | RAB27A. member RAS oncogene family | **-2.799** | | **1.36E-06** | | 0.072 | | 4.95E-01 | |  |
| Cd4 | CD4 | CD4 molecule | **-2.824** | | **1.12E-14** | | **-1.278** | | **1.40E-06** | |  |
| Ckb | CKB | creatine kinase B | **-2.906** | | **1.61E-21** | | **1.407** | | **1.02E-05** | |  |
| Itgb5 | ITGB5 | integrin subunit beta 5 | **-2.914** | | **2.85E-20** | | 0.002 | | 9.93E-01 | |  |
| Stab2 | STAB2 | stabilin 2 | **-2.97** | | **8.38E-04** | | NA | | NA | |  |
| Gas6 | GAS6 | growth arrest specific 6 | **-3.014** | | **7.17E-74** | | -0.645 | | 1.88E-04 | |  |
| Dock2 | DOCK2 | dedicator of cytokinesis 2 | **-3.063** | | **2.16E-20** | | **-1.4** | | **1.16E-06** | |  |
| Cd93 | CD93 | CD93 molecule | **-3.174** | | **1.79E-50** | | 0.019 | | 9.79E-01 | |  |
| Mrc1 | MRC1 | mannose receptor C-type 1 | **-3.179** | | **4.95E-27** | | NA | | NA | |  |
| S100a9 | S100A9 | S100 calcium binding protein A9 | **-3.365** | | **5.19E-03** | | **-3.713** | | **2.40E-05** | |  |
| Sirpb1b | SIRPB1 | signal regulatory protein beta 1 | **-3.403** | | **5.97E-22** | | -0.422 | | 5.20E-04 | |  |
| Cx3cl1 | CX3CL1 | C-X3-C motif chemokine ligand 1 | **-3.477** | | **1.32E-04** | | NA | | NA | |  |
| Mertk | MERTK | MER proto-oncogene. tyrosine kinase | **-3.498** | | **1.20E-19** | | -0.71 | | 8.52E-04 | |  |
| Tlr9 | TLR9 | toll like receptor 9 | **-3.755** | | **1.24E-30** | | NA | | NA | |  |
| Pparg | PPARG | peroxisome proliferator activated receptor gamma | **-3.815** | | **9.15E-30** | | 0.307 | | 8.52E-02 | |  |
| Cyp2s1 | CYP2S1 | cytochrome P450 family 2 subfamily S member 1 | **-4.033** | | **1.10E-06** | | **-1.162** | | **3.46E-05** | |  |
| Activation of phagocytes | | | | | | | | | | | |
| Il1a | IL1A | interleukin 1 alpha | | **9.307** | | **2.85E-222** | | **6.543** | | **1.43E-08** | |
| Csf3 | CSF3 | colony stimulating factor 3 | | **9.086** | | **1.58E-52** | | **1.385** | | **3.75E-05** | |
| Ptgs2 | PTGS2 | prostaglandin-endoperoxide synthase 2 | | **8.664** | | **4.97E-113** | | **4.006** | | **1.95E-05** | |
| Met | MET | MET proto-oncogene. receptor tyrosine kinase | | **7.953** | | **3.43E-201** | | **1.742** | | **1.05E-05** | |
| Ptges | PTGES | prostaglandin E synthase | | **7.764** | | **1.94E-43** | | **-1.006** | | **8.68E-04** | |
| Il1b | IL1B | interleukin 1 beta | | **7.704** | | **7.83E-23** | | **2.474** | | **7.42E-07** | |
| Il6 | IL6 | interleukin 6 | | **7.634** | | **2.72E-22** | | **6.014** | | **6.61E-08** | |
| Il19 | IL19 | interleukin 19 | | **7.603** | | **4.65E-18** | | **6.406** | | **1.36E-08** | |
| S100a8 | S100A8 | S100 calcium binding protein A8 | | **7.377** | | **2.15E-89** | | **-4.124** | | **1.18E-05** | |
| Cxcl2 | CXCL3 | C-X-C motif chemokine ligand 3 | | **6.888** | | **1.71E-17** | | 0.191 | | 1.07E-01 | |
| Saa1 | SAA1 | serum amyloid A1 | | **6.811** | | **2.75E-16** | | NA | | NA | |
| Inhba | INHBA | inhibin beta A subunit | | **6.679** | | **3.02E-53** | | NA | | NA | |
| Il1rn | IL1RN | interleukin 1 receptor antagonist | | **6.515** | | **3.81E-176** | | **3.941** | | **1.35E-06** | |
| Nos2 | NOS2 | nitric oxide synthase 2 | | **6.355** | | **1.21E-09** | | NA | | NA | |
| Edn1 | EDN1 | endothelin 1 | | **6.33** | | **5.70E-26** | | 1.159 | | 9.09E-05 | |
| Slc7a2 | SLC7A2 | solute carrier family 7 member 2 | | **6.219** | | **1.89E-95** | | NA | | NA | |
| Slpi | SLPI | secretory leukocyte peptidase inhibitor | | **5.956** | | **5.72E-19** | | NA | | NA | |
| Hamp | Hamp/Hamp2 | hepcidin antimicrobial peptide | | **5.664** | | **1.02E-08** | | NA | | NA | |
| Adora2b | ADORA2B | adenosine A2b receptor | | **5.632** | | **9.01E-136** | | -0.391 | | 1.40E-02 | |
| Lcn2 | LCN2 | lipocalin 2 | | **5.578** | | **4.55E-11** | | -0.077 | | 5.47E-01 | |
| Socs1 | SOCS1 | suppressor of cytokine signaling 1 | | **5.407** | | **4.33E-46** | | **1.15** | | **5.85E-05** | |
| Tnf | TNF | tumor necrosis factor | | **5.2** | | **5.17E-77** | | **3.336** | | **7.92E-05** | |
| Clec4e | CLEC4E | C-type lectin domain family 4 member E | | **5.157** | | **9.73E-08** | | **1.178** | | **2.24E-05** | |
| Saa3 | Saa3 | serum amyloid A 3 | | **5.077** | | **1.26E-06** | | NA | | NA | |
| Mir125a | mir-10 | microRNA 100 | | **4.054** | | **3.78E-04** | | -0.053 | | 7.18E-01 | |
| Ifi205 | IFI16 | interferon gamma inducible protein 16 | | **4.051** | | **5.94E-61** | | 0.544 | | 5.86E-03 | |
| Cd40 | CD40 | CD40 molecule | | **4.035** | | **9.00E-59** | | **1.229** | | **1.87E-05** | |
| Ahr | AHR | aryl hydrocarbon receptor | | **3.976** | | **1.31E-20** | | 0.309 | | 3.38E-02 | |
| Itga1 | ITGA1 | integrin subunit alpha 1 | | **3.956** | | **4.45E-12** | | **2.582** | | **6.55E-07** | |
| Ccl5 | CCL5 | C-C motif chemokine ligand 5 | | **3.917** | | **1.69E-10** | | **1.453** | | **6.84E-04** | |
| Src | SRC | SRC proto-oncogene. non-receptor tyrosine kinase | | **3.85** | | **2.93E-22** | | **2.341** | | **2.13E-07** | |
| Hmox1 | HMOX1 | heme oxygenase 1 | | **3.722** | | **3.37E-93** | | **-1.424** | | **4.92E-06** | |
| Fpr1 | FPR1 | formyl peptide receptor 1 | | **3.666** | | **1.18E-03** | | **-1.858** | | **1.12E-06** | |
| Mirlet7e | let-7 | microRNA let-7a-1 | | **3.642** | | **2.10E-03** | | 0.145 | | 1.06E-01 | |
| Il10 | IL10 | interleukin 10 | | **3.568** | | **1.42E-59** | | **1.929** | | **3.52E-05** | |
| Cxcl5 | CXCL6 | C-X-C motif chemokine ligand 6 | | **3.514** | | **6.52E-11** | | 0.582 | | 4.29E-04 | |
| Cxcl10 | CXCL10 | C-X-C motif chemokine ligand 10 | | **3.487** | | **1.09E-04** | | NA | | NA | |
| Fpr2 | FPR2 | formyl peptide receptor 2 | | **3.427** | | **3.96E-03** | | 0.534 | | 1.45E-04 | |
| Mir146 | mir-146 | microRNA 146a | | **3.389** | | **5.19E-03** | | 0.05 | | 7.80E-01 | |
| Jak2 | JAK2 | Janus kinase 2 | | **3.362** | | **1.54E-71** | | -0.198 | | 1.34E-01 | |
| Fas | FAS | Fas cell surface death receptor | | **3.204** | | **6.59E-05** | | NA | | NA | |
| Csf2 | CSF2 | colony stimulating factor 2 | | **3.14** | | **1.16E-02** | | **3.814** | | **9.01E-06** | |
| Pf4 | PF4 | platelet factor 4 | | **3.13** | | **2.76E-48** | | NA | | NA | |
| Lta | LTA | lymphotoxin alpha | | **3.106** | | **1.92E-03** | | 0.245 | | 4.64E-02 | |
| Hc | C5 | complement C5 | | **2.795** | | **2.88E-02** | | -0.55 | | 2.76E-04 | |
| Cebpb | CEBPB | CCAAT/enhancer binding protein beta | | **2.768** | | **3.05E-28** | | 0.332 | | 5.43E-03 | |
| Tnfrsf1b | TNFRSF1B | TNF receptor superfamily member 1B | | **2.736** | | **1.22E-38** | | 0.875 | | 2.12E-03 | |
| Cd200 | CD200 | CD200 molecule | | **2.692** | | **8.12E-28** | | 0.186 | | 8.65E-02 | |
| Camp | CAMP | cathelicidin antimicrobial peptide | | **2.677** | | **8.79E-03** | | -0.293 | | 8.35E-03 | |
| Ifnb1 | IFNB1 | interferon beta 1 | | **2.673** | | **3.87E-02** | | NA | | NA | |
| Notch1 | NOTCH1 | notch 1 | | **2.611** | | **1.09E-38** | | -0.962 | | 1.85E-03 | |
| Pla2g5 | PLA2G5 | phospholipase A2 group V | | **2.588** | | **7.69E-03** | | NA | | NA | |
| Msr1 | MSR1 | macrophage scavenger receptor 1 | | **2.437** | | **3.36E-59** | | NA | | NA | |
| Ccl22 | CCL22 | C-C motif chemokine ligand 22 | | **2.409** | | **3.02E-04** | | **1.048** | | **4.38E-06** | |
| Snca | SNCA | synuclein alpha | | **2.404** | | **1.72E-02** | | **1.564** | | **7.40E-05** | |
| Cd14 | CD14 | CD14 molecule | | **2.32** | | **7.36E-21** | | -0.236 | | 7.25E-01 | |
| Slc11a1 | SLC11A1 | solute carrier family 11 member 1 | | **2.316** | | **1.79E-34** | | **-1.139** | | **1.24E-03** | |
| H2-Q7 | HLA-A | major histocompatibility complex. class I. A | | **2.265** | | **1.74E-22** | | 0.368 | | 2.67E-01 | |
| Ccl2 | Ccl2 | chemokine (C-C motif) ligand 2 | | **2.255** | | **5.73E-12** | | NA | | NA | |
| Cd24a | Cd24a | CD24a antigen | | **2.246** | | **3.61E-03** | | NA | | NA | |
| Cd1d1 | CD1D | CD1d molecule | | **2.191** | | **1.81E-11** | | **-3.53** | | **7.41E-08** | |
| Tnfrsf9 | TNFRSF9 | TNF receptor superfamily member 9 | | **2.189** | | **2.86E-04** | | 0.938 | | 1.22E-05 | |
| Icam1 | ICAM1 | intercellular adhesion molecule 1 | | **2.138** | | **1.48E-03** | | **1.319** | | **5.39E-05** | |
| Nfil3 | NFIL3 | nuclear factor. interleukin 3 regulated | | **2.122** | | **3.12E-23** | | **-1.808** | | **7.44E-07** | |
| Itga5 | ITGA5 | integrin subunit alpha 5 | | **1.996** | | **2.89E-29** | | **0.999** | | **7.08E-04** | |
| Lgals9 | LGALS9B | galectin 9B | | **1.973** | | **2.66E-27** | | NA | | NA | |
| Ccl4 | CCL4 | C-C motif chemokine ligand 4 | | **1.919** | | **3.46E-21** | | NA | | NA | |
| Stat1 | STAT1 | signal transducer and activator of transcription 1 | | **1.854** | | **3.69E-12** | | **-1.447** | | **6.77E-03** | |
| Il4ra | IL4R | interleukin 4 receptor | | **1.84** | | **8.82E-40** | | -0.149 | | 1.67E-01 | |
| Tnfrsf10b | TNFRSF10A | TNF receptor superfamily member 10a | | **1.768** | | **9.57E-07** | | 0.249 | | 1.06E-02 | |
| Cybb | CYBB | cytochrome b-245 beta chain | | **1.765** | | **3.11E-07** | | 0.19 | | 2.85E-01 | |
| Ccr7 | CCR7 | C-C motif chemokine receptor 7 | | **1.705** | | **2.59E-02** | | **5.55** | | **7.66E-08** | |
| Thbs1 | THBS1 | thrombospondin 1 | | **1.613** | | **8.43E-23** | | 0.292 | | 7.83E-01 | |
| Hck | HCK | HCK proto-oncogene. Src family tyrosine kinase | | **1.561** | | **3.80E-19** | | **1.842** | | **1.87E-05** | |
| Tlr2 | TLR2 | toll like receptor 2 | | **1.556** | | **1.25E-02** | | 0.316 | | 1.11E-02 | |
| Gja1 | GJA1 | gap junction protein alpha 1 | | **1.549** | | **1.66E-13** | | NA | | NA | |
| Lcp2 | LCP2 | lymphocyte cytosolic protein 2 | | **1.54** | | **9.11E-15** | | 0.454 | | 2.64E-02 | |
| Syk | SYK | spleen associated tyrosine kinase | | **1.491** | | **1.74E-22** | | -0.2 | | 1.06E-01 | |
| Sbno2 | SBNO2 | strawberry notch homolog 2 | | **1.489** | | **7.00E-22** | | -0.199 | | 1.76E-01 | |
| Casp1 | CASP1 | caspase 1 | | **1.403** | | **1.42E-11** | | **1.666** | | **3.90E-06** | |
| Lgals3bp | LGALS3BP | galectin 3 binding protein | | **1.389** | | **9.84E-13** | | -0.378 | | 5.48E-02 | |
| Prkce | PRKCE | protein kinase C epsilon | | **1.378** | | **1.14E-04** | | NA | | NA | |
| Trpm4 | TRPM4 | transient receptor potential cation channel subfamily M member 4 | | **1.374** | | **6.02E-12** | | -0.836 | | 3.36E-05 | |
| Clcn7 | CLCN7 | chloride voltage-gated channel 7 | | **1.372** | | **1.34E-20** | | **1.131** | | **3.03E-05** | |
| Mif | MIF | macrophage migration inhibitory factor (glycosylation-inhibiting factor) | | **1.367** | | **3.53E-23** | | -0.241 | | 2.29E-01 | |
| Osm | OSM | oncostatin M | | **1.319** | | **3.07E-09** | | **1.894** | | **4.00E-04** | |
| Il1r1 | IL1R1 | interleukin 1 receptor type 1 | | **1.216** | | **4.88E-02** | | 0.348 | | 1.74E-03 | |
| Treml2 | TREML2 | triggering receptor expressed on myeloid cells like 2 | | **1.196** | | **3.56E-03** | | NA | | NA | |
| Bid | BID | BH3 interacting domain death agonist | | **1.114** | | **4.72E-06** | | 0.819 | | 5.76E-05 | |
| Rabgef1 | RABGEF1 | RAB guanine nucleotide exchange factor 1 | | **1.114** | | **8.77E-14** | | 0.098 | | 2.78E-01 | |
| Ptger2 | PTGER2 | prostaglandin E receptor 2 | | **1.093** | | **1.01E-04** | | 0.668 | | 2.48E-03 | |
| Ulbp1 | ULBP1 | UL16 binding protein 1 | | **-1.028** | | **4.99E-04** | | -0.041 | | 7.85E-01 | |
| Lrp1 | LRP1 | LDL receptor related protein 1 | | **-1.059** | | **4.17E-05** | | -0.834 | | 1.13E-04 | |
| Cerk | CERK | ceramide kinase | | **-1.159** | | **5.82E-16** | | -0.401 | | 2.35E-02 | |
| P2rx7 | P2RX7 | purinergic receptor P2X 7 | | **-1.208** | | **8.56E-06** | | **2.948** | | **5.35E-05** | |
| Lgals3 | LGALS3 | galectin 3 | | **-1.215** | | **7.76E-10** | | 0.936 | | 8.72E-04 | |
| Apoe | APOE | apolipoprotein E | | **-1.261** | | **5.90E-15** | | NA | | NA | |
| Ptger4 | PTGER4 | prostaglandin E receptor 4 | | **-1.266** | | **2.12E-04** | | 0.485 | | 1.76E-01 | |
| Rarres2 | RARRES2 | retinoic acid receptor responder 2 | | **-1.279** | | **1.82E-05** | | NA | | NA | |
| Rora | RORA | RAR related orphan receptor A | | **-1.292** | | **1.26E-05** | | NA | | NA | |
| Prnp | PRNP | prion protein | | **-1.302** | | **1.77E-12** | | -0.132 | | 4.10E-01 | |
| Tyrobp | TYROBP | TYRO protein tyrosine kinase binding protein | | **-1.303** | | **4.86E-31** | | **-1.629** | | **1.30E-05** | |
| Tpt1 | TPT1 | tumor protein. translationally-controlled 1 | | **-1.354** | | **1.28E-18** | | -0.68 | | 3.60E-01 | |
| Il18 | IL18 | interleukin 18 | | **-1.358** | | **2.47E-03** | | -0.424 | | 7.07E-03 | |
| Cd300ld | CD300LD | CD300 molecule like family member d | | **-1.385** | | **2.80E-24** | | NA | | NA | |
| Ltbp1 | LTBP1 | latent transforming growth factor beta binding protein 1 | | **-1.643** | | **2.29E-03** | | NA | | NA | |
| Metrnl | METRNL | meteorin like. glial cell differentiation regulator | | **-1.664** | | **2.01E-25** | | -0.377 | | 2.31E-02 | |
| Shpk | SHPK | sedoheptulokinase | | **-1.68** | | **5.53E-05** | | -0.265 | | 1.09E-01 | |
| Tnfaip8l2 | TNFAIP8L2 | TNF alpha induced protein 8 like 2 | | **-1.7** | | **1.47E-29** | | 0.158 | | 2.01E-01 | |
| Tspan32 | TSPAN32 | tetraspanin 32 | | **-1.733** | | **5.32E-07** | | **-3.719** | | **1.03E-07** | |
| Tnfsf12 | TNFSF12 | tumor necrosis factor superfamily member 12 | | **-1.797** | | **1.40E-23** | | -0.884 | | 1.63E-04 | |
| Cd300lb | CD300LB | CD300 molecule like family member b | | **-1.815** | | **1.13E-31** | | **-1.19** | | **3.00E-06** | |
| Havcr2 | HAVCR2 | hepatitis A virus cellular receptor 2 | | **-1.815** | | **3.47E-06** | | 0.649 | | 2.55E-02 | |
| C5ar1 | C5AR1 | complement C5a receptor 1 | | **-1.943** | | **9.62E-17** | | **-1.549** | | **2.09E-05** | |
| Fn1 | FN1 | fibronectin 1 | | **-1.95** | | **1.80E-02** | | NA | | NA | |
| Igf1 | IGF1 | insulin like growth factor 1 | | **-1.988** | | **7.16E-35** | | NA | | NA | |
| Abhd12 | ABHD12 | abhydrolase domain containing 12 | | **-1.992** | | **1.57E-19** | | 0.068 | | 5.37E-01 | |
| Trem2 | TREM2 | triggering receptor expressed on myeloid cells 2 | | **-2.026** | | **7.70E-21** | | NA | | NA | |
| Kitl | KITLG | KIT ligand | | **-2.035** | | **6.29E-03** | | NA | | NA | |
| Pros1 | PROS1 | protein S (alpha) | | **-2.172** | | **4.41E-23** | | -0.022 | | 8.73E-01 | |
| Rapgef3 | RAPGEF3 | Rap guanine nucleotide exchange factor 3 | | **-2.194** | | **1.30E-08** | | NA | | NA | |
| Il18rap | IL18RAP | interleukin 18 receptor accessory protein | | **-2.394** | | **1.37E-02** | | -0.218 | | 2.28E-02 | |
| Ptger3 | PTGER3 | prostaglandin E receptor 3 | | **-2.406** | | **2.31E-02** | | 0.153 | | 9.53E-02 | |
| Rhoh | RHOH | ras homolog family member H | | **-2.409** | | **1.29E-16** | | 0.531 | | 1.84E-04 | |
| Angpt2 | ANGPT2 | angiopoietin 2 | | **-2.413** | | **3.97E-07** | | NA | | NA | |
| Anxa1 | ANXA1 | annexin A1 | | **-2.447** | | **1.40E-51** | | -0.468 | | 6.67E-02 | |
| Maf | MAF | MAF bZIP transcription factor | | **-2.499** | | **9.61E-64** | | -0.647 | | 5.29E-03 | |
| Kit | KIT | KIT proto-oncogene receptor tyrosine kinase | | **-2.538** | | **4.13E-13** | | NA | | NA | |
| Cd300a | CD300A | CD300a molecule | | **-2.55** | | **1.79E-22** | | **-1.196** | | **7.74E-06** | |
| Hgf | HGF | hepatocyte growth factor | | **-2.566** | | **5.64E-17** | | NA | | NA | |
| Tnfsf13 | TNFSF13 | tumor necrosis factor superfamily member 13 | | **-2.661** | | **1.21E-08** | | NA | | NA | |
| Clec7a | CLEC7A | C-type lectin domain family 7 member A | | **-2.776** | | **1.84E-38** | | -0.503 | | 4.31E-04 | |
| Tlr8 | TLR8 | toll like receptor 8 | | **-2.796** | | **2.88E-76** | | **1.004** | | **7.38E-03** | |
| Rab27a | RAB27A | RAB27A. member RAS oncogene family | | **-2.799** | | **1.36E-06** | | 0.072 | | 4.95E-01 | |
| Cd4 | CD4 | CD4 molecule | | **-2.824** | | **1.12E-14** | | **-1.278** | | **1.40E-06** | |
| Hpse | HPSE | heparanase | | **-2.855** | | **1.86E-33** | | -0.433 | | 2.08E-02 | |
| Ccr2 | CCR2 | C-C motif chemokine receptor 2 | | **-2.876** | | **1.33E-15** | | -0.226 | | 2.17E-02 | |
| Cd93 | CD93 | CD93 molecule | | **-3.174** | | **1.79E-50** | | 0.019 | | 9.79E-01 | |
| S100a9 | S100A9 | S100 calcium binding protein A9 | | **-3.365** | | **5.19E-03** | | **-3.713** | | **2.40E-05** | |
| Cnr2 | CNR2 | cannabinoid receptor 2 | | **-3.431** | | **4.98E-19** | | NA | | NA | |
| Cx3cl1 | CX3CL1 | C-X3-C motif chemokine ligand 1 | | **-3.477** | | **1.32E-04** | | NA | | NA | |
| Mertk | MERTK | MER proto-oncogene. tyrosine kinase | | **-3.498** | | **1.20E-19** | | -0.71 | | 8.52E-04 | |
| Cfh | CFH | complement factor H | | **-3.522** | | **1.72E-48** | | NA | | NA | |
| Tlr9 | TLR9 | toll like receptor 9 | | **-3.755** | | **1.24E-30** | | NA | | NA | |
| Cx3cr1 | CX3CR1 | C-X3-C motif chemokine receptor 1 | | **-6.34** | | **4.64E-47** | | -0.674 | | 3.91E-04 | |

| **Supplementary Table S3 Proteins identified by LnMS/MS in BMMs stimulated with *B. burgdorferi* or left unstimulated**  Those proteins represented by at least 2 peptides are included. Proteins differentially regulated are indicated (upregulated: shaded orange; downregulated: shaded green). | | | | |  |
| --- | --- | --- | --- | --- | --- |
|  |  |  |  |  | |
| **Accession** | **Description** | **Unique peptides** | **Log(Ratio Borr/Unst)** | **ANOVA** | |
| PGH2_MOUSE | Prostaglandin G/H synthase 2 | 2 | 4.845 | 2.65E-06 | |
| IRG1_MOUSE | Cis-aconitate decarboxylase | 4 | 4.215 | 4.48E-04 | |
| CTR2_MOUSE | Cationic amino acid transporter 2 | 3 | 3.036 | 5.78E-03 | |
| CLC4E_MOUSE | C-type lectin domain family 4 member E | 3 | 2.907 | 6.38E-04 | |
| GBP2_MOUSE | Guanylate-binding protein 1 | 5 | 2.683 | 6.24E-04 | |
| SQSTM_MOUSE | Sequestosome-1 | 6 | 2.668 | 1.67E-03 | |
| IL1B_MOUSE | Interleukin-1 beta | 4 | 2.407 | 4.79E-03 | |
| DEOC_MOUSE | Deoxyribose-phosphate aldolase | 2 | 2.347 | 1.18E-02 | |
| FCGR2_MOUSE | Low affinity immunoglobulin gamma Fc region receptor II | 2 | 2.211 | 7.22E-03 | |
| HMOX1_MOUSE | Heme oxygenase 1 | 5 | 2.061 | 2.46E-03 | |
| ICAM1_MOUSE | Intercellular adhesion molecule 1 | 4 | 1.673 | 6.87E-03 | |
| DDX21_MOUSE | Nucleolar RNA helicase 2 | 3 | 1.509 | 5.48E-03 | |
| PRDX5_MOUSE | Peroxiredoxin-5, mitochondrial | 9 | 1.437 | 9.02E-03 | |
| VPS4B_MOUSE | Vacuolar protein sorting-associated protein 4B | 2 | 1.261 | 1.54E-02 | |
| CD14_MOUSE | Monocyte differentiation antigen CD14 | 7 | 1.246 | 6.25E-03 | |
| ACSL1_MOUSE | Long-chain-fatty-acid--CoA ligase 1 | 12 | 1.210 | 2.80E-03 | |
| 4F2_MOUSE | 4F2 cell-surface antigen heavy chain | 6 | 1.135 | 6.33E-03 | |
| MYO1F_MOUSE | Unconventional myosin-If | 5 | -1.080 | 3.01E-02 | |
| CD36_MOUSE | Platelet glycoprotein 4 | 4 | -1.110 | 6.80E-03 | |
| NPC1_MOUSE | Niemann-Pick C1 protein | 3 | -1.126 | 4.35E-02 | |
| AGAL_MOUSE | Alpha-galactosidase A | 2 | -1.133 | 4.46E-02 | |
| LKHA4_MOUSE | Leukotriene A-4 hydrolase | 6 | -1.138 | 9.15E-03 | |
| AP3B1_MOUSE | AP-3 complex subunit beta-1 | 2 | -1.147 | 4.69E-03 | |
| STOM_MOUSE | Erythrocyte band 7 integral membrane protein | 4 | -1.150 | 1.17E-02 | |
| LRP1_MOUSE | Prolow-density lipoprotein receptor-related protein 1 | 15 | -1.166 | 3.84E-02 | |
| CSF1R_MOUSE | Macrophage colony-stimulating factor 1 receptor | 3 | -1.199 | 3.12E-02 | |
| SCMC1_MOUSE | Calcium-binding mitochondrial carrier protein SCaMC-1 | 2 | -1.218 | 9.82E-03 | |
| ACL6A_MOUSE | Actin-like protein 6A | 2 | -1.268 | 3.67E-02 | |
| LGMN_MOUSE | Legumain | 3 | -1.303 | 2.37E-02 | |
| PP2BA_MOUSE | Serine/threonine-protein phosphatase 2B catalytic subunit alpha isoform | 2 | -1.349 | 2.60E-03 | |
| CD180_MOUSE | CD180 antigen | 2 | -1.349 | 1.65E-02 | |
| PLST_MOUSE | Plastin-3 | 4 | -1.418 | 4.58E-02 | |
| WASP_MOUSE | Wiskott-Aldrich syndrome protein homolog | 4 | -1.665 | 4.06E-02 | |
| NIBAN_MOUSE | Protein Niban | 2 | -1.951 | 3.86E-02 | |
| LIPL_MOUSE | Lipoprotein lipase | 2 | -2.430 | 1.55E-02 | |
| PEBP1_MOUSE | Phosphatidylethanolamine-binding protein 1 | 2 | 2.607 | 1.05E-01 | |
| TXNL1_MOUSE | Thioredoxin-like protein 1 | 2 | 2.355 | 1.05E-01 | |
| TMA16_MOUSE | Translation machinery-associated protein 16 | 2 | 2.103 | 6.10E-02 | |
| MYO1C_MOUSE | Unconventional myosin-Ic | 2 | 2.087 | 2.75E-01 | |
| RS17_MOUSE | 40S ribosomal protein S17 | 2 | 1.834 | 1.95E-01 | |
| PROSC_MOUSE | Proline synthase co-transcribed bacterial homolog protein | 2 | 1.417 | 3.03E-01 | |
| SUCB1_MOUSE | Succinyl-CoA ligase [ADP-forming] subunit beta, mitochondrial | 3 | 1.328 | 5.97E-02 | |
| ASSY_MOUSE | Argininosuccinate synthase | 2 | 1.108 | 8.46E-02 | |
| IFM3_MOUSE | Interferon-induced transmembrane protein 3 | 2 | 1.083 | 1.17E-01 | |
| TBA4A_MOUSE | Tubulin alpha-4A chain | 12 | 1.059 | 1.46E-01 | |
| SODM_MOUSE | Superoxide dismutase [Mn], mitochondrial | 2 | 1.042 | 9.68E-02 | |
| RL21_MOUSE | 60S ribosomal protein L21 OS=Mus musculus GN=Rpl21 PE=1 SV=3 | 2 | 1.041 | 2.19E-01 | |
| HNRL2_MOUSE | Heterogeneous nuclear ribonucleoprotein U-like protein 2 | 2 | 1.031 | 1.59E-01 | |
| SMRC2_MOUSE | SWI/SNF complex subunit SMARCC2 | 2 | 1.018 | 8.13E-02 | |
| PTN1_MOUSE | Tyrosine-protein phosphatase non-receptor type 1 | 4 | 1.003 | 1.03E-01 | |
| FLII_MOUSE | Protein flightless-1 homolog | 4 | 0.988 | 5.40E-01 | |
| XPP1_MOUSE | Xaa-Pro aminopeptidase 1 | 2 | 0.960 | 2.16E-01 | |
| LRC25_MOUSE | Leucine-rich repeat-containing protein 25 | 4 | 0.944 | 7.71E-02 | |
| VATD_MOUSE | V-type proton ATPase subunit D | 3 | 0.941 | 3.90E-01 | |
| ACSL4_MOUSE | Long-chain-fatty-acid--CoA ligase 4 | 4 | 0.879 | 2.03E-02 | |
| TLR2_MOUSE | Toll-like receptor 2 | 3 | 0.862 | 8.20E-02 | |
| AGRE1_MOUSE | Adhesion G protein-coupled receptor E1 | 3 | 0.851 | 1.73E-01 | |
| EFHD2_MOUSE | EF-hand domain-containing protein D2 | 6 | 0.839 | 2.04E-01 | |
| ODP2_MOUSE | Dihydrolipoyllysine-residue acetyltransferase component of pyruvate dehydrogenase complex, mitochondrial | 2 | 0.830 | 1.82E-01 | |
| CY24B_MOUSE | Cytochrome b-245 heavy chain | 2 | 0.816 | 1.07E-01 | |
| E2AK2_MOUSE | Interferon-induced, double-stranded RNA-activated protein kinase | 3 | 0.811 | 1.68E-01 | |
| MANF_MOUSE | Mesencephalic astrocyte-derived neurotrophic factor | 2 | 0.807 | 1.31E-01 | |
| SH3K1_MOUSE | SH3 domain-containing kinase-binding protein 1 | 2 | 0.775 | 3.29E-01 | |
| EHD1_MOUSE | EH domain-containing protein 1 | 6 | 0.771 | 4.01E-02 | |
| AMPD3_MOUSE | AMP deaminase 3 | 3 | 0.765 | 1.08E-01 | |
| TWF1_MOUSE | Twinfilin-1 | 2 | 0.754 | 1.48E-01 | |
| XDH_MOUSE | Xanthine dehydrogenase/oxidase | 2 | 0.753 | 3.46E-01 | |
| PLEK_MOUSE | Pleckstrin | 2 | 0.726 | 2.02E-01 | |
| PSD11_MOUSE | 26S proteasome non-ATPase regulatory subunit 11 | 5 | 0.725 | 9.64E-02 | |
| IDE_MOUSE | Insulin-degrading enzyme | 2 | 0.712 | 2.79E-01 | |
| PNPH_MOUSE | Purine nucleoside phosphorylase | 8 | 0.668 | 7.26E-02 | |
| NCF1_MOUSE | Neutrophil cytosol factor 1 | 2 | 0.667 | 2.27E-01 | |
| AT1B3_MOUSE | Sodium/potassium-transporting ATPase subunit beta-3 | 2 | 0.661 | 2.34E-01 | |
| FYB_MOUSE | FYN-binding protein | 3 | 0.659 | 3.06E-02 | |
| IF5A1_MOUSE | Eukaryotic translation initiation factor 5A-1 | 2 | 0.653 | 1.37E-01 | |
| NAMPT_MOUSE | Nicotinamide phosphoribosyltransferase | 6 | 0.647 | 1.30E-01 | |
| TRXR1_MOUSE | Thioredoxin reductase 1, cytoplasmic | 5 | 0.631 | 5.88E-02 | |
| IFIT3_MOUSE | Interferon-induced protein with tetratricopeptide repeats 3 | 4 | 0.629 | 2.99E-01 | |
| EIF3D_MOUSE | Eukaryotic translation initiation factor 3 subunit D | 3 | 0.627 | 1.16E-01 | |
| DCTN2_MOUSE | Dynactin subunit 2 | 2 | 0.611 | 1.01E-01 | |
| IFIT1_MOUSE | Interferon-induced protein with tetratricopeptide repeats 1 | 3 | 0.601 | 9.74E-02 | |
| PSB3_MOUSE | Proteasome subunit beta type-3 | 2 | 0.598 | 7.31E-03 | |
| NP1L4_MOUSE | Nucleosome assembly protein 1-like 4 | 3 | 0.579 | 5.27E-01 | |
| OSTF1_MOUSE | Osteoclast-stimulating factor 1 | 3 | 0.573 | 3.10E-01 | |
| PROF1_MOUSE | Profilin-1 | 4 | 0.569 | 2.63E-01 | |
| TOIP1_MOUSE | Torsin-1A-interacting protein 1 | 2 | 0.569 | 7.91E-02 | |
| MP2K1_MOUSE | Dual specificity mitogen-activated protein kinase kinase 1 | 2 | 0.565 | 3.06E-01 | |
| CHM4B_MOUSE | Charged multivesicular body protein 4b | 3 | 0.561 | 1.19E-01 | |
| MIF_MOUSE | Macrophage migration inhibitory factor | 2 | 0.516 | 8.67E-01 | |
| PURA2_MOUSE | Adenylosuccinate synthetase isozyme 2 | 3 | 0.500 | 1.96E-01 | |
| HCLS1_MOUSE | Hematopoietic lineage cell-specific protein | 8 | 0.500 | 1.43E-01 | |
| VASP_MOUSE | Vasodilator-stimulated phosphoprotein | 3 | 0.498 | 2.88E-01 | |
| BLVRB_MOUSE | Flavin reductase (NADPH) | 5 | 0.496 | 4.31E-01 | |
| RS28_MOUSE | 40S ribosomal protein S28 | 2 | 0.494 | 3.04E-01 | |
| DSCR3_MOUSE | Down syndrome critical region protein 3 homolog | 2 | 0.492 | 2.86E-01 | |
| LA_MOUSE | Lupus La protein homolog | 4 | 0.490 | 4.18E-01 | |
| RL12_MOUSE | 60S ribosomal protein L12 | 3 | 0.486 | 4.01E-01 | |
| TPSN_MOUSE | Tapasin | 3 | 0.484 | 2.60E-01 | |
| XPO2_MOUSE | Exportin-2 | 4 | 0.481 | 3.01E-01 | |
| PRS6A_MOUSE | 26S protease regulatory subunit 6A | 4 | 0.460 | 3.76E-01 | |
| LEG3_MOUSE | Galectin-3 | 4 | 0.454 | 5.17E-01 | |
| FPPS_MOUSE | Farnesyl pyrophosphate synthase | 3 | 0.452 | 1.48E-01 | |
| PGM1_MOUSE | Phosphoglucomutase-1 | 3 | 0.430 | 2.56E-01 | |
| LEG9_MOUSE | Galectin-9 | 2 | 0.423 | 4.22E-01 | |
| RL13_MOUSE | 60S ribosomal protein L13 | 5 | 0.407 | 4.84E-01 | |
| ASNS_MOUSE | Asparagine synthetase [glutamine-hydrolyzing] | 2 | 0.398 | 5.52E-01 | |
| RS29_MOUSE | 40S ribosomal protein S29 | 2 | 0.395 | 5.96E-01 | |
| TPM4_MOUSE | Tropomyosin alpha-4 chain | 9 | 0.375 | 6.88E-02 | |
| TCTP_MOUSE | Translationally-controlled tumor protein | 2 | 0.369 | 7.86E-01 | |
| MOES_MOUSE | Moesin | 19 | 0.360 | 2.20E-01 | |
| AB1IP_MOUSE | Amyloid beta A4 precursor protein-binding family B member 1-interacting protein | 2 | 0.353 | 1.73E-01 | |
| PTGR1_MOUSE | Prostaglandin reductase 1 | 4 | 0.344 | 5.43E-01 | |
| DNPEP_MOUSE | Aspartyl aminopeptidase | 3 | 0.336 | 1.81E-01 | |
| BROX_MOUSE | BRO1 domain-containing protein BROX | 2 | 0.332 | 2.64E-01 | |
| 6PGL_MOUSE | 6-phosphogluconolactonase | 2 | 0.326 | 4.25E-01 | |
| COPB_MOUSE | Coatomer subunit beta | 5 | 0.321 | 4.32E-01 | |
| BLMH_MOUSE | Bleomycin hydrolase | 3 | 0.320 | 8.07E-01 | |
| DCTN1_MOUSE | Dynactin subunit 1 | 4 | 0.310 | 4.72E-01 | |
| CD44_MOUSE | CD44 antigen | 4 | 0.297 | 1.44E-01 | |
| SYIC_MOUSE | Isoleucine--tRNA ligase, cytoplasmic | 4 | 0.292 | 3.84E-01 | |
| PABP1_MOUSE | Polyadenylate-binding protein 1 | 14 | 0.287 | 6.69E-02 | |
| RS2_MOUSE | 40S ribosomal protein S2 | 6 | 0.283 | 7.12E-01 | |
| PRDX6_MOUSE | Peroxiredoxin-6 | 6 | 0.281 | 4.71E-01 | |
| IF4A1_MOUSE | Eukaryotic initiation factor 4A-I | 10 | 0.280 | 2.85E-01 | |
| GNA13_MOUSE | Guanine nucleotide-binding protein subunit alpha-13 | 3 | 0.280 | 3.38E-01 | |
| SH3L3_MOUSE | SH3 domain-binding glutamic acid-rich-like protein 3 | 2 | 0.275 | 6.63E-01 | |
| TPIS_MOUSE | Triosephosphate isomerase | 5 | 0.266 | 3.47E-01 | |
| COR1B_MOUSE | Coronin-1B | 6 | 0.265 | 5.54E-01 | |
| NONO_MOUSE | Non-POU domain-containing octamer-binding protein | 4 | 0.262 | 2.25E-01 | |
| RS20_MOUSE | 40S ribosomal protein S20 | 3 | 0.259 | 3.13E-01 | |
| COPB2_MOUSE | Coatomer subunit beta' | 6 | 0.259 | 4.34E-01 | |
| TCPZ_MOUSE | T-complex protein 1 subunit zeta | 10 | 0.252 | 2.30E-01 | |
| EZRI_MOUSE | Ezrin | 9 | 0.249 | 2.40E-01 | |
| MVP_MOUSE | Major vault protein | 13 | 0.247 | 2.05E-01 | |
| IF4G1_MOUSE | Eukaryotic translation initiation factor 4 gamma 1 | 4 | 0.240 | 4.64E-01 | |
| PARP1_MOUSE | Poly [ADP-ribose] polymerase 1 | 2 | 0.236 | 7.64E-01 | |
| KSYK_MOUSE | Tyrosine-protein kinase SYK | 3 | 0.235 | 2.61E-01 | |
| RAB32_MOUSE | Ras-related protein Rab-32 | 3 | 0.235 | 3.29E-01 | |
| CX6B1_MOUSE | Cytochrome c oxidase subunit 6B1 | 2 | 0.228 | 4.85E-01 | |
| EIF3G_MOUSE | Eukaryotic translation initiation factor 3 subunit G | 2 | 0.226 | 7.08E-01 | |
| RS13_MOUSE | 40S ribosomal protein S13 | 2 | 0.225 | 5.55E-01 | |
| ITAM_MOUSE | Integrin alpha-M | 11 | 0.225 | 4.27E-01 | |
| HCK_MOUSE | Tyrosine-protein kinase HCK | 6 | 0.225 | 2.42E-01 | |
| RL4_MOUSE | 60S ribosomal protein L4 | 9 | 0.223 | 5.19E-01 | |
| RS19_MOUSE | 40S ribosomal protein S19 | 5 | 0.222 | 4.97E-01 | |
| RLA2_MOUSE | 60S acidic ribosomal protein P2 | 3 | 0.221 | 7.54E-01 | |
| IF4E_MOUSE | Eukaryotic translation initiation factor 4E | 2 | 0.221 | 4.36E-01 | |
| GSH0_MOUSE | Glutamate--cysteine ligase regulatory subunit | 2 | 0.219 | 4.41E-01 | |
| PSMD2_MOUSE | 26S proteasome non-ATPase regulatory subunit 2 | 9 | 0.216 | 5.72E-01 | |
| KPCD_MOUSE | Protein kinase C delta type | 7 | 0.215 | 3.19E-01 | |
| IDHP_MOUSE | Isocitrate dehydrogenase [NADP], mitochondrial | 5 | 0.209 | 5.16E-01 | |
| SNP23_MOUSE | Synaptosomal-associated protein 23 | 2 | 0.209 | 6.68E-01 | |
| RS4X_MOUSE | 40S ribosomal protein S4, X isoform | 6 | 0.202 | 2.22E-01 | |
| CAND1_MOUSE | Cullin-associated NEDD8-dissociated protein 1 | 6 | 0.197 | 4.48E-01 | |
| HMOX2_MOUSE | Heme oxygenase 2 | 2 | 0.197 | 5.33E-01 | |
| RS11_MOUSE | 40S ribosomal protein S11 | 8 | 0.196 | 7.16E-01 | |
| PGK1_MOUSE | Phosphoglycerate kinase 1 | 16 | 0.193 | 5.69E-01 | |
| EIF3F_MOUSE | Eukaryotic translation initiation factor 3 subunit F | 2 | 0.186 | 6.16E-01 | |
| EIF3C_MOUSE | Eukaryotic translation initiation factor 3 subunit C | 4 | 0.178 | 1.32E-01 | |
| TBB6_MOUSE | Tubulin beta-6 chain | 8 | 0.175 | 4.73E-01 | |
| ANXA6_MOUSE | Annexin A6 | 15 | 0.174 | 6.89E-01 | |
| CAPR1_MOUSE | Caprin-1 | 4 | 0.168 | 8.52E-01 | |
| RL28_MOUSE | 60S ribosomal protein L28 | 2 | 0.166 | 8.18E-01 | |
| PSMD4_MOUSE | 26S proteasome non-ATPase regulatory subunit 4 | 3 | 0.165 | 1.32E-01 | |
| LBR_MOUSE | Lamin-B receptor | 2 | 0.164 | 2.37E-01 | |
| ESTD_MOUSE | S-formylglutathione hydrolase | 6 | 0.161 | 7.02E-01 | |
| GPDM_MOUSE | Glycerol-3-phosphate dehydrogenase, mitochondrial | 3 | 0.159 | 5.04E-01 | |
| LEG1_MOUSE | Galectin-1 | 4 | 0.157 | 8.38E-01 | |
| PIPNA_MOUSE | Phosphatidylinositol transfer protein alpha isoform | 4 | 0.155 | 7.26E-01 | |
| ODO2_MOUSE | Dihydrolipoyllysine-residue succinyltransferase component of 2-oxoglutarate dehydrogenase complex, mitochondrial | 2 | 0.151 | 5.27E-01 | |
| AP2M1_MOUSE | AP-2 complex subunit mu | 4 | 0.151 | 5.43E-01 | |
| VATH_MOUSE | V-type proton ATPase subunit H | 5 | 0.148 | 5.97E-01 | |
| LAMP1_MOUSE | Lysosome-associated membrane glycoprotein 1 | 4 | 0.146 | 7.10E-01 | |
| RL7A_MOUSE | 60S ribosomal protein L7a | 8 | 0.139 | 9.74E-01 | |
| RS16_MOUSE | 40S ribosomal protein S16 | 5 | 0.138 | 5.95E-01 | |
| PP1A_MOUSE | Serine/threonine-protein phosphatase PP1-alpha catalytic subunit | 4 | 0.137 | 9.48E-01 | |
| RL8_MOUSE | 60S ribosomal protein L8 | 3 | 0.127 | 7.39E-01 | |
| AN32B_MOUSE | Acidic leucine-rich nuclear phosphoprotein 32 family member B | 2 | 0.125 | 6.60E-01 | |
| LASP1_MOUSE | LIM and SH3 domain protein 1 | 4 | 0.121 | 6.96E-01 | |
| PSB2_MOUSE | Proteasome subunit beta type-2 | 2 | 0.117 | 5.67E-01 | |
| PRS8_MOUSE | 26S protease regulatory subunit 8 | 3 | 0.105 | 7.83E-01 | |
| BCAP_MOUSE | Phosphoinositide 3-kinase adapter protein 1 | 3 | 0.101 | 5.58E-01 | |
| RS3A_MOUSE | 40S ribosomal protein S3a | 8 | 0.101 | 8.46E-01 | |
| DDX3X_MOUSE | ATP-dependent RNA helicase DDX3X | 5 | 0.101 | 6.12E-01 | |
| SYDC_MOUSE | Aspartate--tRNA ligase, cytoplasmic | 5 | 0.100 | 5.35E-01 | |
| SC11A_MOUSE | Signal peptidase complex catalytic subunit SEC11A | 3 | 0.098 | 6.66E-01 | |
| LPPRC_MOUSE | Leucine-rich PPR motif-containing protein, mitochondrial | 4 | 0.096 | 5.92E-01 | |
| ETFB_MOUSE | Electron transfer flavoprotein subunit beta | 3 | 0.096 | 9.30E-01 | |
| FLNB_MOUSE | Filamin-B | 8 | 0.090 | 6.19E-01 | |
| PEPD_MOUSE | Xaa-Pro dipeptidase | 3 | 0.090 | 9.77E-01 | |
| G6PD1_MOUSE | Glucose-6-phosphate 1-dehydrogenase X | 14 | 0.087 | 8.46E-01 | |
| ERLN2_MOUSE | Erlin-2 | 3 | 0.086 | 6.08E-01 | |
| HNRH1_MOUSE | Heterogeneous nuclear ribonucleoprotein H | 4 | 0.084 | 9.64E-01 | |
| LRC59_MOUSE | Leucine-rich repeat-containing protein 59 | 5 | 0.082 | 7.42E-01 | |
| SQRD_MOUSE | Sulfide:quinone oxidoreductase, mitochondrial | 2 | 0.080 | 7.14E-01 | |
| RHG17_MOUSE | Rho GTPase-activating protein 17 | 4 | 0.080 | 6.88E-01 | |
| TPD52_MOUSE | Tumor protein D52 | 3 | 0.080 | 8.95E-01 | |
| ROA1_MOUSE | Heterogeneous nuclear ribonucleoprotein A1 | 3 | 0.078 | 7.46E-01 | |
| RL23A_MOUSE | 60S ribosomal protein L23a | 6 | 0.077 | 9.91E-01 | |
| MARCS_MOUSE | Myristoylated alanine-rich C-kinase substrate | 4 | 0.077 | 9.94E-01 | |
| RL5_MOUSE | 60S ribosomal protein L5 | 6 | 0.075 | 8.27E-01 | |
| CPNS1_MOUSE | Calpain small subunit 1 | 2 | 0.070 | 8.65E-01 | |
| MACF1_MOUSE | Microtubule-actin cross-linking factor 1 | 3 | 0.070 | 7.46E-01 | |
| HSP7C_MOUSE | Heat shock cognate 71 kDa protein | 21 | 0.065 | 7.28E-01 | |
| PSME2_MOUSE | Proteasome activator complex subunit 2 | 5 | 0.064 | 8.78E-01 | |
| DDX17_MOUSE | Probable ATP-dependent RNA helicase DDX17 | 8 | 0.063 | 3.65E-01 | |
| GLYG_MOUSE | Glycogenin-1 | 2 | 0.060 | 7.73E-01 | |
| LYN_MOUSE | Tyrosine-protein kinase Lyn | 6 | 0.060 | 7.81E-01 | |
| SAR1B_MOUSE | GTP-binding protein SAR1b | 2 | 0.050 | 9.01E-01 | |
| CASP1_MOUSE | Caspase-1 | 5 | 0.045 | 8.07E-01 | |
| PPCE_MOUSE | Prolyl endopeptidase | 7 | 0.045 | 8.17E-01 | |
| SC31A_MOUSE | Protein transport protein Sec31A | 5 | 0.043 | 9.20E-01 | |
| RAB6A_MOUSE | Ras-related protein Rab-6A | 3 | 0.042 | 9.81E-01 | |
| IF4G2_MOUSE | Eukaryotic translation initiation factor 4 gamma 2 | 2 | 0.040 | 9.96E-01 | |
| U520_MOUSE | U5 small nuclear ribonucleoprotein 200 kDa helicase | 2 | 0.040 | 7.06E-01 | |
| ITB1_MOUSE | Integrin beta-1 | 8 | 0.038 | 8.67E-01 | |
| RS3_MOUSE | 40S ribosomal protein S3 | 9 | 0.037 | 8.36E-01 | |
| COPZ1_MOUSE | Coatomer subunit zeta-1 | 2 | 0.036 | 8.43E-01 | |
| NPM_MOUSE | Nucleophosmin | 2 | 0.035 | 8.87E-01 | |
| PSMD6_MOUSE | 26S proteasome non-ATPase regulatory subunit 6 | 3 | 0.029 | 9.21E-01 | |
| RL22_MOUSE | 60S ribosomal protein L22 | 2 | 0.026 | 9.88E-01 | |
| RS15A_MOUSE | 40S ribosomal protein S15a | 2 | 0.025 | 8.13E-01 | |
| ABR_MOUSE | Active breakpoint cluster region-related protein | 3 | 0.025 | 9.34E-01 | |
| PSB1_MOUSE | Proteasome subunit beta type-1 | 4 | 0.024 | 7.92E-01 | |
| RL27A_MOUSE | 60S ribosomal protein L27a | 3 | 0.023 | 9.77E-01 | |
| RN213_MOUSE | E3 ubiquitin-protein ligase RNF213 | 5 | 0.021 | 9.52E-01 | |
| SMD3_MOUSE | Small nuclear ribonucleoprotein Sm D3 | 2 | 0.019 | 8.99E-01 | |
| TAGL2_MOUSE | Transgelin-2 | 9 | 0.017 | 9.70E-01 | |
| DDX58_MOUSE | Probable ATP-dependent RNA helicase DDX58 | 3 | 0.014 | 8.33E-01 | |
| PSA5_MOUSE | Proteasome subunit alpha type-5 | 3 | 0.013 | 9.87E-01 | |
| RTN4_MOUSE | Reticulon-4 | 4 | 0.009 | 8.91E-01 | |
| HNRPQ_MOUSE | Heterogeneous nuclear ribonucleoprotein Q | 7 | 0.009 | 9.77E-01 | |
| HNRPC_MOUSE | Heterogeneous nuclear ribonucleoproteins C1/C2 | 6 | 0.008 | 8.94E-01 | |
| ANXA7_MOUSE | Annexin A7 | 4 | 0.008 | 9.67E-01 | |
| KCD12_MOUSE | BTB/POZ domain-containing protein KCTD12 | 6 | 0.007 | 9.47E-01 | |
| PP2AA_MOUSE | Serine/threonine-protein phosphatase 2A catalytic subunit alpha isoform | 2 | 0.005 | 7.41E-01 | |
| YBOX1_MOUSE | Nuclease-sensitive element-binding protein 1 | 3 | 0.003 | 8.58E-01 | |
| VATE1_MOUSE | V-type proton ATPase subunit E 1 | 7 | 0.002 | 9.33E-01 | |
| MAP4_MOUSE | Microtubule-associated protein 4 | 4 | -0.007 | 9.48E-01 | |
| RS26_MOUSE | 40S ribosomal protein S26 | 2 | -0.011 | 9.14E-01 | |
| NDKB_MOUSE | Nucleoside diphosphate kinase B | 5 | -0.011 | 8.24E-01 | |
| RS12_MOUSE | 40S ribosomal protein S12 | 4 | -0.011 | 9.74E-01 | |
| NICA_MOUSE | Nicastrin | 2 | -0.017 | 9.72E-01 | |
| EF2_MOUSE | Elongation factor 2 | 19 | -0.017 | 9.71E-01 | |
| TBB4B_MOUSE | Tubulin beta-4B chain | 11 | -0.019 | 9.98E-01 | |
| RS10_MOUSE | 40S ribosomal protein S10 | 2 | -0.020 | 9.22E-01 | |
| NDKA_MOUSE | Nucleoside diphosphate kinase A | 5 | -0.020 | 8.98E-01 | |
| RL31_MOUSE | 60S ribosomal protein L31 | 3 | -0.022 | 7.84E-01 | |
| HSP74_MOUSE | Heat shock 70 kDa protein 4 | 13 | -0.024 | 8.98E-01 | |
| SYG_MOUSE | Glycine--tRNA ligase | 3 | -0.026 | 9.79E-01 | |
| BAX_MOUSE | Apoptosis regulator BAX | 3 | -0.027 | 8.34E-01 | |
| GDIR2_MOUSE | Rho GDP-dissociation inhibitor 2 | 3 | -0.028 | 9.94E-01 | |
| PGAM1_MOUSE | Phosphoglycerate mutase 1 | 6 | -0.030 | 8.38E-01 | |
| OAS1A_MOUSE | 2'-5'-oligoadenylate synthase 1A | 3 | -0.031 | 7.97E-01 | |
| RL26_MOUSE | 60S ribosomal protein L26 | 3 | -0.032 | 7.81E-01 | |
| HXK2_MOUSE | Hexokinase-2 | 8 | -0.040 | 7.15E-01 | |
| C1TM_MOUSE | Monofunctional C1-tetrahydrofolate synthase, mitochondrial | 3 | -0.042 | 9.64E-01 | |
| EIF3B_MOUSE | Eukaryotic translation initiation factor 3 subunit B | 4 | -0.048 | 8.20E-01 | |
| TM9S4_MOUSE | Transmembrane 9 superfamily member 4 | 2 | -0.049 | 9.13E-01 | |
| EIF3L_MOUSE | Eukaryotic translation initiation factor 3 subunit L | 5 | -0.050 | 9.15E-01 | |
| TENS3_MOUSE | Tensin-3 | 2 | -0.053 | 9.10E-01 | |
| ERF3A_MOUSE | Eukaryotic peptide chain release factor GTP-binding subunit ERF3A | 2 | -0.055 | 7.31E-01 | |
| PUR2_MOUSE | Trifunctional purine biosynthetic protein adenosine-3 | 2 | -0.057 | 7.85E-01 | |
| RL24_MOUSE | 60S ribosomal protein L24 | 4 | -0.057 | 7.80E-01 | |
| PRDX1_MOUSE | Peroxiredoxin-1 | 11 | -0.058 | 6.98E-01 | |
| TBB5_MOUSE | Tubulin beta-5 chain | 10 | -0.060 | 8.68E-01 | |
| VAV_MOUSE | Proto-oncogene vav | 3 | -0.060 | 7.60E-01 | |
| SGPL1_MOUSE | Sphingosine-1-phosphate lyase 1 | 6 | -0.064 | 9.34E-01 | |
| RRBP1_MOUSE | Ribosome-binding protein 1 | 11 | -0.064 | 7.75E-01 | |
| MYH9_MOUSE | Myosin-9 | 56 | -0.064 | 9.23E-01 | |
| ARPC2_MOUSE | Actin-related protein 2/3 complex subunit 2 | 8 | -0.065 | 6.73E-01 | |
| UGPA_MOUSE | UTP--glucose-1-phosphate uridylyltransferase | 2 | -0.065 | 7.14E-01 | |
| NCF4_MOUSE | Neutrophil cytosol factor 4 | 6 | -0.065 | 8.68E-01 | |
| COPD_MOUSE | Coatomer subunit delta | 7 | -0.065 | 8.28E-01 | |
| EF1G_MOUSE | Elongation factor 1-gamma | 7 | -0.067 | 7.49E-01 | |
| H2B1B_MOUSE | Histone H2B type 1-B | 6 | -0.068 | 8.67E-01 | |
| RS18_MOUSE | 40S ribosomal protein S18 | 6 | -0.070 | 7.10E-01 | |
| SCOT1_MOUSE | Succinyl-CoA:3-ketoacid coenzyme A transferase 1, mitochondrial | 4 | -0.071 | 5.77E-01 | |
| 2ABA_MOUSE | Serine/threonine-protein phosphatase 2A 55 kDa regulatory subunit B alpha isoform | 2 | -0.072 | 8.97E-01 | |
| LRRF1_MOUSE | Leucine-rich repeat flightless-interacting protein 1 | 2 | -0.073 | 6.46E-01 | |
| PSME1_MOUSE | Proteasome activator complex subunit 1 | 7 | -0.075 | 7.22E-01 | |
| SF3B3_MOUSE | Splicing factor 3B subunit 3 | 3 | -0.076 | 7.97E-01 | |
| RAGP1_MOUSE | Ran GTPase-activating protein 1 | 2 | -0.076 | 9.33E-01 | |
| RS27A_MOUSE | Ubiquitin-40S ribosomal protein S27a | 3 | -0.080 | 8.23E-01 | |
| PICAL_MOUSE | Phosphatidylinositol-binding clathrin assembly protein | 3 | -0.080 | 7.11E-01 | |
| TCPB_MOUSE | T-complex protein 1 subunit beta | 6 | -0.081 | 7.31E-01 | |
| HPCL1_MOUSE | Hippocalcin-like protein 1 | 5 | -0.085 | 8.05E-01 | |
| RS8_MOUSE | 40S ribosomal protein S8 | 6 | -0.087 | 6.93E-01 | |
| RAP1B_MOUSE | Ras-related protein Rap-1b | 5 | -0.088 | 6.86E-01 | |
| CAB39_MOUSE | Calcium-binding protein 39 | 3 | -0.089 | 6.75E-01 | |
| TCPQ_MOUSE | T-complex protein 1 subunit theta | 14 | -0.089 | 6.07E-01 | |
| SND1_MOUSE | Staphylococcal nuclease domain-containing protein 1 | 7 | -0.096 | 5.52E-01 | |
| HNRPF_MOUSE | Heterogeneous nuclear ribonucleoprotein F | 4 | -0.096 | 7.29E-01 | |
| ADT2_MOUSE | ADP/ATP translocase 2 | 9 | -0.096 | 7.83E-01 | |
| RL32_MOUSE | 60S ribosomal protein L32 | 3 | -0.101 | 7.49E-01 | |
| PSA4_MOUSE | Proteasome subunit alpha type-4 | 2 | -0.103 | 9.94E-01 | |
| GSHR_MOUSE | Glutathione reductase, mitochondrial | 3 | -0.103 | 4.24E-01 | |
| DIAP1_MOUSE | Protein diaphanous homolog 1 | 4 | -0.103 | 5.02E-01 | |
| SKAP2_MOUSE | Src kinase-associated phosphoprotein 2 | 3 | -0.104 | 7.59E-01 | |
| RACK1_MOUSE | Receptor of activated protein C kinase 1 | 8 | -0.104 | 3.92E-01 | |
| PSMD1_MOUSE | 26S proteasome non-ATPase regulatory subunit 1 | 7 | -0.105 | 5.77E-01 | |
| PRS10_MOUSE | 26S protease regulatory subunit 10B | 5 | -0.106 | 8.79E-01 | |
| FLNA_MOUSE | Filamin-A | 50 | -0.108 | 6.45E-01 | |
| ARPC3_MOUSE | Actin-related protein 2/3 complex subunit 3 | 4 | -0.109 | 7.33E-01 | |
| ADRM1_MOUSE | Proteasomal ubiquitin receptor ADRM1 | 2 | -0.109 | 5.51E-01 | |
| EI3JA_MOUSE | Eukaryotic translation initiation factor 3 subunit J-A | 2 | -0.110 | 7.41E-01 | |
| PAIRB_MOUSE | Plasminogen activator inhibitor 1 RNA-binding protein | 2 | -0.111 | 2.72E-01 | |
| RLA0_MOUSE | 60S acidic ribosomal protein P0 | 6 | -0.112 | 5.90E-01 | |
| RL7_MOUSE | 60S ribosomal protein L7 | 8 | -0.113 | 3.54E-01 | |
| ANM1_MOUSE | Protein arginine N-methyltransferase 1 | 3 | -0.113 | 9.01E-01 | |
| DNJC7_MOUSE | DnaJ homolog subfamily C member 7 | 2 | -0.115 | 7.28E-01 | |
| RAB18_MOUSE | Ras-related protein Rab-18 | 4 | -0.116 | 7.22E-01 | |
| CALX_MOUSE | Calnexin | 9 | -0.118 | 5.63E-01 | |
| EF1B_MOUSE | Elongation factor 1-beta | 4 | -0.119 | 6.34E-01 | |
| VINC_MOUSE | Vinculin | 5 | -0.120 | 7.58E-01 | |
| H4_MOUSE | Histone H4 | 6 | -0.121 | 7.46E-01 | |
| LDHA_MOUSE | L-lactate dehydrogenase A chain | 9 | -0.122 | 6.59E-01 | |
| IF2A_MOUSE | Eukaryotic translation initiation factor 2 subunit 1 | 6 | -0.123 | 1.47E-01 | |
| ARF1_MOUSE | ADP-ribosylation factor 1 | 5 | -0.123 | 6.48E-01 | |
| PSB6_MOUSE | Proteasome subunit beta type-6 | 2 | -0.123 | 4.91E-01 | |
| SEP11_MOUSE | Septin-11 | 3 | -0.125 | 9.68E-01 | |
| IF2B_MOUSE | Eukaryotic translation initiation factor 2 subunit 2 | 6 | -0.125 | 8.32E-01 | |
| NACA_MOUSE | Nascent polypeptide-associated complex subunit alpha | 2 | -0.126 | 7.48E-01 | |
| PPIA_MOUSE | Peptidyl-prolyl cis-trans isomerase A | 7 | -0.129 | 5.89E-01 | |
| ACTN4_MOUSE | Alpha-actinin-4 | 14 | -0.131 | 6.27E-01 | |
| UBE2K_MOUSE | Ubiquitin-conjugating enzyme E2 K | 2 | -0.132 | 8.50E-01 | |
| LIS1_MOUSE | Platelet-activating factor acetylhydrolase IB subunit alpha | 2 | -0.134 | 5.84E-01 | |
| TMED5_MOUSE | Transmembrane emp24 domain-containing protein 5 | 2 | -0.134 | 7.15E-01 | |
| ATPB_MOUSE | ATP synthase subunit beta, mitochondrial | 12 | -0.134 | 5.31E-01 | |
| ACADL_MOUSE | Long-chain specific acyl-CoA dehydrogenase, mitochondrial | 4 | -0.135 | 3.03E-01 | |
| FCERG_MOUSE | High affinity immunoglobulin epsilon receptor subunit gamma | 3 | -0.135 | 8.39E-01 | |
| PSA3_MOUSE | Proteasome subunit alpha type-3 | 3 | -0.136 | 5.87E-01 | |
| PSA7_MOUSE | Proteasome subunit alpha type-7 O | 4 | -0.141 | 6.26E-01 | |
| GRP78_MOUSE | 78 kDa glucose-regulated protein | 17 | -0.141 | 6.55E-01 | |
| SPTB2_MOUSE | Spectrin beta chain, non-erythrocytic 1 | 4 | -0.142 | 8.02E-01 | |
| NIBL1_MOUSE | Niban-like protein 1 | 7 | -0.142 | 5.65E-01 | |
| RAB8B_MOUSE | Ras-related protein Rab-8B | 7 | -0.146 | 6.15E-01 | |
| RS25_MOUSE | 40S ribosomal protein S25 | 3 | -0.148 | 6.01E-01 | |
| SERC_MOUSE | Phosphoserine aminotransferase | 4 | -0.148 | 2.17E-01 | |
| ERO1A_MOUSE | ERO1-like protein alpha | 3 | -0.149 | 8.29E-01 | |
| EF1D_MOUSE | Elongation factor 1-delta | 3 | -0.149 | 4.68E-01 | |
| AK1A1_MOUSE | Alcohol dehydrogenase [NADP(+)] | 7 | -0.152 | 5.09E-01 | |
| PSA6_MOUSE | Proteasome subunit alpha type-6 | 3 | -0.154 | 5.49E-01 | |
| RL3_MOUSE | 60S ribosomal protein L3 | 4 | -0.156 | 5.61E-01 | |
| G3BP1_MOUSE | Ras GTPase-activating protein-binding protein 1 | 2 | -0.158 | 4.00E-01 | |
| RL30_MOUSE | 60S ribosomal protein L30 | 3 | -0.158 | 4.05E-01 | |
| RL10_MOUSE | 60S ribosomal protein L10 | 2 | -0.160 | 9.72E-01 | |
| AT2A2_MOUSE | Sarcoplasmic/endoplasmic reticulum calcium ATPase 2 | 10 | -0.160 | 5.13E-01 | |
| RL6_MOUSE | 60S ribosomal protein L6 | 7 | -0.160 | 5.85E-01 | |
| RSU1_MOUSE | Ras suppressor protein 1 | 3 | -0.162 | 4.68E-01 | |
| ILK_MOUSE | Integrin-linked protein kinase | 3 | -0.163 | 4.10E-01 | |
| ADHX_MOUSE | Alcohol dehydrogenase class-3 | 2 | -0.166 | 4.40E-01 | |
| TECR_MOUSE | Very-long-chain enoyl-CoA reductase | 2 | -0.167 | 9.52E-01 | |
| SP16H_MOUSE | FACT complex subunit SPT16 | 2 | -0.167 | 6.01E-01 | |
| NCF2_MOUSE | Neutrophil cytosol factor 2 | 3 | -0.167 | 6.41E-01 | |
| IMB1_MOUSE | Importin subunit beta-1 | 6 | -0.169 | 9.97E-01 | |
| RL34_MOUSE | 60S ribosomal protein L34 | 2 | -0.169 | 5.25E-01 | |
| CAZA2_MOUSE | F-actin-capping protein subunit alpha-2 | 3 | -0.174 | 4.78E-01 | |
| FAK2_MOUSE | Protein-tyrosine kinase 2-beta | 4 | -0.178 | 3.68E-01 | |
| IPO5_MOUSE | Importin-5 | 7 | -0.178 | 5.00E-01 | |
| TADBP_MOUSE | TAR DNA-binding protein 43 | 3 | -0.178 | 3.63E-01 | |
| UGGG1_MOUSE | UDP-glucose:glycoprotein glucosyltransferase 1 | 7 | -0.178 | 4.59E-01 | |
| HNRPK_MOUSE | Heterogeneous nuclear ribonucleoprotein K | 12 | -0.179 | 5.76E-01 | |
| DDX5_MOUSE | Probable ATP-dependent RNA helicase DDX5 | 11 | -0.179 | 2.32E-01 | |
| RS6_MOUSE | 40S ribosomal protein S6 | 3 | -0.181 | 3.83E-01 | |
| ALDOA_MOUSE | Fructose-bisphosphate aldolase A | 10 | -0.182 | 5.18E-01 | |
| K1C10_MOUSE | Keratin, type I cytoskeletal 10 | 4 | -0.185 | 5.24E-01 | |
| IMA4_MOUSE | Importin subunit alpha-4 | 2 | -0.185 | 3.37E-01 | |
| SARNP_MOUSE | SAP domain-containing ribonucleoprotein | 3 | -0.185 | 6.79E-01 | |
| GMFB_MOUSE | Glia maturation factor beta | 2 | -0.186 | 6.50E-01 | |
| 1433Z_MOUSE | 14-3-3 protein zeta/delta | 9 | -0.186 | 6.23E-01 | |
| G3P_MOUSE | Glyceraldehyde-3-phosphate dehydrogenase | 8 | -0.186 | 4.16E-01 | |
| FRIL1_MOUSE | Ferritin light chain 1 | 2 | -0.189 | 2.24E-01 | |
| FUMH_MOUSE | Fumarate hydratase, mitochondrial | 3 | -0.190 | 5.51E-01 | |
| SAC1_MOUSE | Phosphatidylinositide phosphatase SAC1 | 3 | -0.190 | 5.07E-01 | |
| CATA_MOUSE | Catalase | 11 | -0.190 | 7.26E-01 | |
| RS27L_MOUSE | 40S ribosomal protein S27-like | 2 | -0.191 | 5.14E-01 | |
| COTL1_MOUSE | Coactosin-like protein | 3 | -0.191 | 7.68E-01 | |
| ENOA_MOUSE | Alpha-enolase | 14 | -0.192 | 3.96E-01 | |
| SYAC_MOUSE | Alanine--tRNA ligase, cytoplasmic | 2 | -0.193 | 7.52E-01 | |
| ARF5_MOUSE | ADP-ribosylation factor 5 | 5 | -0.196 | 6.98E-01 | |
| AN32E_MOUSE | Acidic leucine-rich nuclear phosphoprotein 32 family member E | 2 | -0.197 | 5.52E-01 | |
| CISY_MOUSE | Citrate synthase, mitochondrial | 6 | -0.199 | 4.20E-01 | |
| 6PGD_MOUSE | 6-phosphogluconate dehydrogenase, decarboxylating | 11 | -0.201 | 4.97E-01 | |
| SERA_MOUSE | D-3-phosphoglycerate dehydrogenase | 6 | -0.202 | 5.72E-01 | |
| CATB_MOUSE | Cathepsin B | 8 | -0.203 | 4.53E-01 | |
| RRAGC_MOUSE | Ras-related GTP-binding protein C | 3 | -0.204 | 1.49E-01 | |
| DC1L2_MOUSE | Cytoplasmic dynein 1 light intermediate chain 2 | 2 | -0.204 | 5.26E-01 | |
| ROA3_MOUSE | Heterogeneous nuclear ribonucleoprotein A3 | 5 | -0.206 | 3.00E-01 | |
| LAP2B_MOUSE | Lamina-associated polypeptide 2, isoforms beta/delta/epsilon/gamma | 3 | -0.208 | 3.28E-01 | |
| CAPZB_MOUSE | F-actin-capping protein subunit beta | 5 | -0.209 | 4.30E-01 | |
| RHOA_MOUSE | Transforming protein RhoA | 3 | -0.212 | 3.20E-01 | |
| SYK_MOUSE | Lysine--tRNA ligase | 4 | -0.212 | 4.24E-01 | |
| RL27_MOUSE | 60S ribosomal protein L27 | 3 | -0.214 | 4.81E-01 | |
| PYGB_MOUSE | Glycogen phosphorylase, brain form | 4 | -0.215 | 3.96E-01 | |
| DHX9_MOUSE | ATP-dependent RNA helicase A | 3 | -0.216 | 5.73E-01 | |
| ROA2_MOUSE | Heterogeneous nuclear ribonucleoproteins A2/B1 | 9 | -0.217 | 3.49E-01 | |
| SYEP_MOUSE | Bifunctional glutamate/proline--tRNA ligase | 9 | -0.219 | 3.73E-01 | |
| NSF_MOUSE | Vesicle-fusing ATPase | 5 | -0.220 | 3.64E-01 | |
| RAN_MOUSE | GTP-binding nuclear protein Ran | 5 | -0.220 | 4.25E-01 | |
| KINH_MOUSE | Kinesin-1 heavy chain | 5 | -0.221 | 4.09E-01 | |
| TCPA_MOUSE | T-complex protein 1 subunit alpha | 12 | -0.221 | 3.34E-01 | |
| RL35A_MOUSE | 60S ribosomal protein L35a | 2 | -0.222 | 4.54E-01 | |
| IDHC_MOUSE | Isocitrate dehydrogenase [NADP] cytoplasmic | 12 | -0.223 | 5.14E-01 | |
| HS90B_MOUSE | Heat shock protein HSP 90-beta | 20 | -0.223 | 5.95E-01 | |
| PRS4_MOUSE | 26S protease regulatory subunit 4 | 3 | -0.224 | 5.07E-01 | |
| ANFY1_MOUSE | Rabankyrin-5 | 3 | -0.227 | 5.15E-01 | |
| VATC1_MOUSE | V-type proton ATPase subunit C 1 | 4 | -0.228 | 5.19E-01 | |
| TLN1_MOUSE | Talin-1 | 47 | -0.228 | 2.19E-01 | |
| MBB1A_MOUSE | Myb-binding protein 1A | 5 | -0.229 | 4.77E-01 | |
| GSDMD_MOUSE | Gasdermin-D | 3 | -0.233 | 2.65E-01 | |
| PRS7_MOUSE | 26S protease regulatory subunit 7 | 7 | -0.234 | 4.85E-01 | |
| PDC6I_MOUSE | Programmed cell death 6-interacting protein | 9 | -0.235 | 1.18E-01 | |
| EF1A1_MOUSE | Elongation factor 1-alpha 1 | 12 | -0.237 | 3.10E-01 | |
| TCPG_MOUSE | T-complex protein 1 subunit gamma | 14 | -0.239 | 1.25E-01 | |
| CAZA1_MOUSE | F-actin-capping protein subunit alpha-1 | 3 | -0.239 | 2.98E-01 | |
| FA49B_MOUSE | Protein FAM49B | 4 | -0.240 | 4.14E-01 | |
| ACTB_MOUSE | Actin, cytoplasmic 1 | 12 | -0.240 | 3.47E-01 | |
| SWP70_MOUSE | Switch-associated protein 70 | 2 | -0.240 | 7.74E-01 | |
| UBP5_MOUSE | Ubiquitin carboxyl-terminal hydrolase 5 | 3 | -0.241 | 1.95E-01 | |
| RBBP4_MOUSE | Histone-binding protein RBBP4 | 3 | -0.241 | 4.85E-01 | |
| SODC_MOUSE | Superoxide dismutase [Cu-Zn] | 2 | -0.242 | 4.68E-01 | |
| MATR3_MOUSE | Matrin-3 | 3 | -0.243 | 7.18E-01 | |
| RAB1A_MOUSE | Ras-related protein Rab-1A | 6 | -0.243 | 3.47E-01 | |
| STAT1_MOUSE | Signal transducer and activator of transcription 1 | 7 | -0.244 | 4.45E-01 | |
| SDHA_MOUSE | Succinate dehydrogenase [ubiquinone] flavoprotein subunit, mitochondrial | 3 | -0.245 | 9.44E-01 | |
| CALM_MOUSE | Calmodulin | 5 | -0.247 | 5.28E-01 | |
| SYCC_MOUSE | Cysteine--tRNA ligase, cytoplasmic | 2 | -0.248 | 4.47E-01 | |
| RHOG_MOUSE | Rho-related GTP-binding protein RhoG | 7 | -0.249 | 4.05E-01 | |
| UBE2N_MOUSE | Ubiquitin-conjugating enzyme E2 N | 3 | -0.250 | 3.83E-01 | |
| GDIR1_MOUSE | Rho GDP-dissociation inhibitor 1 | 5 | -0.250 | 5.24E-01 | |
| PCBP2_MOUSE | Poly(rC)-binding protein 2 | 6 | -0.251 | 5.08E-01 | |
| F10A1_MOUSE | Hsc70-interacting protein | 6 | -0.252 | 1.57E-01 | |
| VATA_MOUSE | V-type proton ATPase catalytic subunit A | 10 | -0.254 | 4.84E-01 | |
| EIF3M_MOUSE | Eukaryotic translation initiation factor 3 subunit M | 3 | -0.254 | 2.17E-01 | |
| UBP14_MOUSE | Ubiquitin carboxyl-terminal hydrolase 14 | 3 | -0.255 | 3.71E-01 | |
| SAHH_MOUSE | Adenosylhomocysteinase | 5 | -0.255 | 3.24E-01 | |
| VPS29_MOUSE | Vacuolar protein sorting-associated protein 29 | 2 | -0.256 | 4.83E-01 | |
| HXK1_MOUSE | Hexokinase-1 | 6 | -0.258 | 5.56E-01 | |
| AMRP_MOUSE | Alpha-2-macroglobulin receptor-associated protein | 3 | -0.258 | 6.16E-01 | |
| ARP3_MOUSE | Actin-related protein 3 | 9 | -0.259 | 5.44E-01 | |
| ELAV1_MOUSE | ELAV-like protein 1 | 4 | -0.260 | 4.02E-01 | |
| PDLI5_MOUSE | PDZ and LIM domain protein 5 | 2 | -0.261 | 5.60E-01 | |
| MYO1G_MOUSE | Unconventional myosin-Ig | 2 | -0.261 | 6.86E-01 | |
| PSA_MOUSE | Puromycin-sensitive aminopeptidase | 5 | -0.261 | 1.47E-01 | |
| ML12B_MOUSE | Myosin regulatory light chain 12B | 5 | -0.262 | 5.23E-01 | |
| TCPD_MOUSE | T-complex protein 1 subunit delta | 9 | -0.265 | 5.40E-01 | |
| CYB5_MOUSE | Cytochrome b5 | 3 | -0.265 | 5.27E-01 | |
| DX39B_MOUSE | Spliceosome RNA helicase Ddx39b | 5 | -0.268 | 4.54E-01 | |
| 1433G_MOUSE | 14-3-3 protein gamma | 5 | -0.268 | 5.31E-01 | |
| FKBP4_MOUSE | Peptidyl-prolyl cis-trans isomerase FKBP4 | 3 | -0.270 | 4.84E-01 | |
| TR150_MOUSE | Thyroid hormone receptor-associated protein 3 | 2 | -0.270 | 4.61E-01 | |
| DC1L1_MOUSE | Cytoplasmic dynein 1 light intermediate chain 1 | 3 | -0.271 | 9.48E-02 | |
| GBB2_MOUSE | Guanine nucleotide-binding protein G(I)/G(S)/G(T) subunit beta-2 | 4 | -0.274 | 4.25E-01 | |
| ETFA_MOUSE | Electron transfer flavoprotein subunit alpha, mitochondrial | 4 | -0.276 | 4.87E-01 | |
| DHRS1_MOUSE | Dehydrogenase/reductase SDR family member 1 | 2 | -0.280 | 3.86E-01 | |
| GNS_MOUSE | N-acetylglucosamine-6-sulfatase | 6 | -0.282 | 7.29E-01 | |
| SMD2_MOUSE | Small nuclear ribonucleoprotein Sm D2 | 3 | -0.283 | 2.87E-01 | |
| NOP56_MOUSE | Nucleolar protein 56 | 3 | -0.285 | 5.34E-01 | |
| RISC_MOUSE | Retinoid-inducible serine carboxypeptidase | 2 | -0.288 | 4.64E-01 | |
| UAP1L_MOUSE | UDP-N-acetylhexosamine pyrophosphorylase-like protein 1 | 5 | -0.289 | 1.33E-01 | |
| RS9_MOUSE | 40S ribosomal protein S9 | 5 | -0.289 | 6.01E-01 | |
| RPN1_MOUSE | Dolichyl-diphosphooligosaccharide--protein glycosyltransferase subunit 1 | 9 | -0.289 | 5.39E-01 | |
| PUR6_MOUSE | Multifunctional protein ADE2 | 3 | -0.290 | 3.16E-01 | |
| SEPT7_MOUSE | Septin-7 | 3 | -0.291 | 1.14E-01 | |
| SC22B_MOUSE | Vesicle-trafficking protein SEC22b O | 3 | -0.292 | 1.77E-01 | |
| 1433B_MOUSE | 14-3-3 protein beta/alpha | 7 | -0.299 | 4.18E-01 | |
| ENPL_MOUSE | Endoplasmin | 22 | -0.303 | 3.07E-01 | |
| ERAP1_MOUSE | Endoplasmic reticulum aminopeptidase 1 | 3 | -0.304 | 8.41E-01 | |
| RAB14_MOUSE | Ras-related protein Rab-14 | 3 | -0.306 | 3.64E-01 | |
| NAGAB_MOUSE | Alpha-N-acetylgalactosaminidase | 3 | -0.306 | 1.95E-01 | |
| TNPO1_MOUSE | Transportin-1 | 3 | -0.308 | 3.76E-01 | |
| DJB11_MOUSE | DnaJ homolog subfamily B member 11 | 2 | -0.309 | 3.24E-01 | |
| HA1B_MOUSE | H-2 class I histocompatibility antigen, K-B alpha chain | 5 | -0.309 | 7.23E-01 | |
| SEPT9_MOUSE | Septin-9 | 4 | -0.311 | 3.40E-01 | |
| AN32A_MOUSE | Acidic leucine-rich nuclear phosphoprotein 32 family member A | 3 | -0.311 | 2.22E-01 | |
| PFKAP_MOUSE | ATP-dependent 6-phosphofructokinase, platelet type | 6 | -0.313 | 1.61E-01 | |
| FKB15_MOUSE | FK506-binding protein 15 | 3 | -0.313 | 3.85E-01 | |
| BUB3_MOUSE | Mitotic checkpoint protein BUB3 | 3 | -0.314 | 3.95E-01 | |
| ACTN1_MOUSE | Alpha-actinin-1 | 15 | -0.317 | 3.75E-01 | |
| THIKA_MOUSE | 3-ketoacyl-CoA thiolase A, peroxisomal | 7 | -0.319 | 3.09E-01 | |
| ARF4_MOUSE | ADP-ribosylation factor 4 | 4 | -0.322 | 2.59E-01 | |
| FABP5_MOUSE | Fatty acid-binding protein, epidermal | 3 | -0.323 | 8.98E-02 | |
| RL13A_MOUSE | 60S ribosomal protein L13a | 2 | -0.323 | 2.61E-01 | |
| PPIB_MOUSE | Peptidyl-prolyl cis-trans isomerase B | 7 | -0.324 | 2.63E-01 | |
| SSRD_MOUSE | Translocon-associated protein subunit delta | 2 | -0.326 | 1.12E-01 | |
| ACLY_MOUSE | ATP-citrate synthase | 7 | -0.327 | 1.47E-01 | |
| SRSF1_MOUSE | Serine/arginine-rich splicing factor 1 | 3 | -0.327 | 2.70E-01 | |
| UBA1_MOUSE | Ubiquitin-like modifier-activating enzyme 1 | 13 | -0.327 | 9.80E-02 | |
| OTUB1_MOUSE | Ubiquitin thioesterase OTUB1 | 2 | -0.327 | 3.17E-01 | |
| LAMP2_MOUSE | Lysosome-associated membrane glycoprotein 2 | 4 | -0.328 | 8.61E-02 | |
| ARP2_MOUSE | Actin-related protein 2 | 5 | -0.333 | 1.21E-01 | |
| USP9X_MOUSE | Probable ubiquitin carboxyl-terminal hydrolase FAF-X | 5 | -0.339 | 3.18E-01 | |
| AP2A1_MOUSE | AP-2 complex subunit alpha-1 | 2 | -0.340 | 4.38E-01 | |
| RAP2B_MOUSE | Ras-related protein Rap-2b | 3 | -0.341 | 2.62E-01 | |
| SFPQ_MOUSE | Splicing factor, proline- and glutamine-rich | 6 | -0.343 | 2.51E-01 | |
| RL10A_MOUSE | 60S ribosomal protein L10a | 5 | -0.345 | 6.81E-01 | |
| SHPS1_MOUSE | Tyrosine-protein phosphatase non-receptor type substrate 1 | 3 | -0.351 | 9.33E-01 | |
| 2AAA_MOUSE | Serine/threonine-protein phosphatase 2A 65 kDa regulatory subunit A alpha isoform | 8 | -0.351 | 2.00E-01 | |
| RL14_MOUSE | 60S ribosomal protein L14 | 4 | -0.355 | 2.98E-01 | |
| RB22A_MOUSE | Ras-related protein Rab-22A | 3 | -0.359 | 1.83E-01 | |
| DPYL2_MOUSE | Dihydropyrimidinase-related protein 2 | 11 | -0.359 | 2.27E-01 | |
| SYVC_MOUSE | Valine--tRNA ligase | 3 | -0.359 | 4.68E-01 | |
| RL18_MOUSE | 60S ribosomal protein L18 | 5 | -0.360 | 3.51E-01 | |
| DYHC1_MOUSE | Cytoplasmic dynein 1 heavy chain 1 | 29 | -0.361 | 3.32E-01 | |
| HA11_MOUSE | H-2 class I histocompatibility antigen, D-B alpha chain | 7 | -0.364 | 4.47E-01 | |
| DAB2_MOUSE | Disabled homolog 2 | 3 | -0.364 | 1.27E-01 | |
| CALR_MOUSE | Calreticulin | 11 | -0.365 | 1.46E-01 | |
| TBB2A_MOUSE | Tubulin beta-2A chain | 10 | -0.365 | 2.07E-01 | |
| ROAA_MOUSE | Heterogeneous nuclear ribonucleoprotein A/B | 5 | -0.365 | 2.60E-01 | |
| NUCL_MOUSE | Nucleolin | 13 | -0.367 | 1.48E-01 | |
| WDR1_MOUSE | WD repeat-containing protein 1 | 11 | -0.368 | 1.40E-01 | |
| SPRE_MOUSE | Sepiapterin reductase | 2 | -0.369 | 7.87E-01 | |
| SSRA_MOUSE | Translocon-associated protein subunit alpha | 2 | -0.370 | 9.22E-02 | |
| WASF2_MOUSE | Wiskott-Aldrich syndrome protein family member 2 | 4 | -0.373 | 1.42E-01 | |
| TCPH_MOUSE | T-complex protein 1 subunit eta | 11 | -0.373 | 1.91E-01 | |
| SYTC_MOUSE | Threonine--tRNA ligase, cytoplasmic | 2 | -0.374 | 1.73E-01 | |
| UBC12_MOUSE | NEDD8-conjugating enzyme Ubc12 | 3 | -0.376 | 6.07E-01 | |
| OST48_MOUSE | Dolichyl-diphosphooligosaccharide--protein glycosyltransferase 48 kDa subunit | 6 | -0.377 | 2.66E-01 | |
| MK03_MOUSE | Mitogen-activated protein kinase 3 | 7 | -0.378 | 1.18E-01 | |
| RAB5C_MOUSE | Ras-related protein Rab-5C | 4 | -0.378 | 1.72E-01 | |
| RL23_MOUSE | 60S ribosomal protein L23 | 4 | -0.379 | 9.87E-01 | |
| SYFB_MOUSE | Phenylalanine--tRNA ligase beta subunit | 4 | -0.380 | 1.22E-02 | |
| MRC1_MOUSE | Macrophage mannose receptor 1 | 5 | -0.381 | 5.33E-01 | |
| CYFP1_MOUSE | Cytoplasmic FMR1-interacting protein 1 | 10 | -0.381 | 2.16E-01 | |
| CAP1_MOUSE | Adenylyl cyclase-associated protein 1 | 12 | -0.382 | 4.42E-01 | |
| MYL6_MOUSE | Myosin light polypeptide 6 | 6 | -0.383 | 1.37E-01 | |
| 1433E_MOUSE | 14-3-3 protein epsilon | 10 | -0.384 | 1.62E-01 | |
| MPCP_MOUSE | Phosphate carrier protein, mitochondrial | 3 | -0.384 | 4.77E-02 | |
| PERM_MOUSE | Myeloperoxidase | 6 | -0.385 | 1.81E-01 | |
| CD166_MOUSE | CD166 antigen | 4 | -0.386 | 2.01E-01 | |
| RADI_MOUSE | Radixin | 8 | -0.388 | 2.07E-01 | |
| TERA_MOUSE | Transitional endoplasmic reticulum ATPase | 19 | -0.389 | 1.09E-02 | |
| RL15_MOUSE | 60S ribosomal protein L15 | 5 | -0.389 | 1.91E-01 | |
| RSSA_MOUSE | 40S ribosomal protein SA | 6 | -0.390 | 1.24E-01 | |
| RL9_MOUSE | 60S ribosomal protein L9 | 2 | -0.391 | 4.65E-01 | |
| VAT1_MOUSE | Synaptic vesicle membrane protein VAT-1 homolog | 7 | -0.391 | 2.20E-01 | |
| RL11_MOUSE | 60S ribosomal protein L11 | 2 | -0.392 | 2.36E-01 | |
| CORO7_MOUSE | Coronin-7 | 3 | -0.393 | 2.59E-01 | |
| EIF3E_MOUSE | Eukaryotic translation initiation factor 3 subunit E | 4 | -0.394 | 2.00E-01 | |
| ARLY_MOUSE | Argininosuccinate lyase | 3 | -0.394 | 7.89E-02 | |
| MPRD_MOUSE | Cation-dependent mannose-6-phosphate receptor | 3 | -0.395 | 1.38E-01 | |
| PRAF3_MOUSE | PRA1 family protein 3 | 2 | -0.397 | 1.69E-02 | |
| PRDX2_MOUSE | Peroxiredoxin-2 | 4 | -0.401 | 1.84E-01 | |
| RBM39_MOUSE | RNA-binding protein 39 | 2 | -0.402 | 3.40E-01 | |
| ADT1_MOUSE | ADP/ATP translocase 1 | 7 | -0.405 | 6.55E-02 | |
| SET_MOUSE | Protein SET | 3 | -0.406 | 2.40E-01 | |
| PRP19_MOUSE | Pre-mRNA-processing factor 19 | 2 | -0.407 | 2.39E-01 | |
| LYRIC_MOUSE | Protein LYRIC | 2 | -0.407 | 1.44E-01 | |
| APEX1_MOUSE | DNA-(apurinic or apyrimidinic site) lyase | 3 | -0.410 | 2.12E-01 | |
| RL18A_MOUSE | 60S ribosomal protein L18a | 2 | -0.411 | 6.12E-01 | |
| PLD4_MOUSE | Phospholipase D4 | 3 | -0.413 | 7.90E-02 | |
| CAPG_MOUSE | Macrophage-capping protein | 4 | -0.414 | 1.58E-01 | |
| ARPC4_MOUSE | Actin-related protein 2/3 complex subunit 4 | 4 | -0.415 | 7.39E-02 | |
| RAB1B_MOUSE | Ras-related protein Rab-1B | 7 | -0.417 | 1.88E-01 | |
| ATPO_MOUSE | ATP synthase subunit O, mitochondrial | 4 | -0.418 | 2.00E-01 | |
| SYSC_MOUSE | Serine--tRNA ligase, cytoplasmic | 2 | -0.419 | 1.53E-01 | |
| KPYM_MOUSE | Pyruvate kinase PKM | 19 | -0.421 | 8.69E-02 | |
| PTN6_MOUSE | Tyrosine-protein phosphatase non-receptor type 6 | 10 | -0.425 | 2.43E-01 | |
| ODO1_MOUSE | 2-oxoglutarate dehydrogenase, mitochondrial | 4 | -0.425 | 2.34E-02 | |
| PLEC_MOUSE | Plectin | 84 | -0.425 | 6.37E-02 | |
| HXK3_MOUSE | Hexokinase-3 | 13 | -0.427 | 2.00E-01 | |
| HINT1_MOUSE | Histidine triad nucleotide-binding protein 1 | 2 | -0.428 | 1.76E-01 | |
| SNX1_MOUSE | Sorting nexin-1 | 11 | -0.429 | 2.73E-01 | |
| NMT1_MOUSE | Glycylpeptide N-tetradecanoyltransferase 1 | 2 | -0.429 | 2.28E-01 | |
| H15_MOUSE | Histone H1.5 | 6 | -0.430 | 2.48E-01 | |
| VDAC1_MOUSE | Voltage-dependent anion-selective channel protein 1 | 3 | -0.434 | 1.19E-01 | |
| TGM2_MOUSE | Protein-glutamine gamma-glutamyltransferase 2 | 8 | -0.438 | 2.58E-01 | |
| EM55_MOUSE | 55 kDa erythrocyte membrane protein | 4 | -0.442 | 1.25E-01 | |
| RINI_MOUSE | Ribonuclease inhibitor | 16 | -0.442 | 3.51E-02 | |
| RALY_MOUSE | RNA-binding protein Raly | 2 | -0.443 | 1.11E-01 | |
| SNAA_MOUSE | Alpha-soluble NSF attachment protein | 3 | -0.443 | 2.16E-01 | |
| SHIP1_MOUSE | Phosphatidylinositol 3,4,5-trisphosphate 5-phosphatase 1 | 2 | -0.444 | 2.81E-01 | |
| VP26A_MOUSE | Vacuolar protein sorting-associated protein 26A | 3 | -0.444 | 2.18E-01 | |
| GLU2B_MOUSE | Glucosidase 2 subunit beta | 3 | -0.445 | 3.60E-01 | |
| INO1_MOUSE | Inositol-3-phosphate synthase 1 | 2 | -0.447 | 5.93E-01 | |
| GLCM_MOUSE | Glucosylceramidase | 4 | -0.447 | 9.58E-02 | |
| NDUA4_MOUSE | Cytochrome c oxidase subunit NDUFA4 | 2 | -0.448 | 8.49E-02 | |
| COPA_MOUSE | Coatomer subunit alpha | 6 | -0.451 | 1.47E-01 | |
| RHG01_MOUSE | Rho GTPase-activating protein 1 | 3 | -0.453 | 3.57E-01 | |
| RL19_MOUSE | 60S ribosomal protein L19 | 2 | -0.455 | 2.51E-01 | |
| AMPB_MOUSE | Aminopeptidase B | 6 | -0.457 | 1.23E-01 | |
| VAPB_MOUSE | Vesicle-associated membrane protein-associated protein B | 2 | -0.459 | 5.00E-02 | |
| AT1A1_MOUSE | Sodium/potassium-transporting ATPase subunit alpha-1 | 15 | -0.460 | 2.35E-01 | |
| CASP8_MOUSE | Caspase-8 | 4 | -0.461 | 2.66E-01 | |
| PTPRC_MOUSE | Receptor-type tyrosine-protein phosphatase C | 12 | -0.462 | 2.23E-01 | |
| CAN1_MOUSE | Calpain-1 catalytic subunit | 3 | -0.464 | 5.97E-02 | |
| BIEA_MOUSE | Biliverdin reductase A | 6 | -0.467 | 1.84E-01 | |
| FAM21_MOUSE | WASH complex subunit FAM21 | 2 | -0.468 | 5.31E-01 | |
| GNPI1_MOUSE | Glucosamine-6-phosphate isomerase 1 | 4 | -0.470 | 5.17E-03 | |
| PDXK_MOUSE | Pyridoxal kinase | 4 | -0.471 | 1.74E-02 | |
| HS90A_MOUSE | Heat shock protein HSP 90-alpha | 18 | -0.471 | 3.14E-01 | |
| ECHB_MOUSE | Trifunctional enzyme subunit beta, mitochondrial | 4 | -0.472 | 1.42E-01 | |
| LMAN2_MOUSE | Vesicular integral-membrane protein VIP36 | 4 | -0.473 | 1.33E-01 | |
| HPRT_MOUSE | Hypoxanthine-guanine phosphoribosyltransferase | 6 | -0.474 | 1.37E-01 | |
| CLIC1_MOUSE | Chloride intracellular channel protein 1 | 10 | -0.474 | 8.68E-02 | |
| K2C1_MOUSE | Keratin, type II cytoskeletal 1 | 3 | -0.474 | 3.03E-01 | |
| CDC37_MOUSE | Hsp90 co-chaperone Cdc37 | 4 | -0.477 | 4.17E-01 | |
| PDIA4_MOUSE | Protein disulfide-isomerase A4 | 10 | -0.478 | 6.33E-02 | |
| GRP75_MOUSE | Stress-70 protein, mitochondrial | 13 | -0.478 | 1.87E-01 | |
| SNX3_MOUSE | Sorting nexin-3 | 3 | -0.479 | 1.52E-02 | |
| PA2G4_MOUSE | Proliferation-associated protein 2G4 | 6 | -0.480 | 9.49E-02 | |
| TIF1B_MOUSE | Transcription intermediary factor 1-beta | 4 | -0.481 | 1.83E-02 | |
| GSTM1_MOUSE | Glutathione S-transferase Mu 1 | 8 | -0.484 | 8.51E-02 | |
| AHSA1_MOUSE | Activator of 90 kDa heat shock protein ATPase homolog 1 | 6 | -0.486 | 6.47E-02 | |
| DOCK2_MOUSE | Dedicator of cytokinesis protein 2 | 7 | -0.487 | 1.16E-01 | |
| TPP2_MOUSE | Tripeptidyl-peptidase 2 | 7 | -0.490 | 1.75E-01 | |
| IDH3A_MOUSE | Isocitrate dehydrogenase [NAD] subunit alpha, mitochondrial | 4 | -0.492 | 5.32E-02 | |
| STXB2_MOUSE | Syntaxin-binding protein 2 | 2 | -0.496 | 2.04E-01 | |
| RUVB2_MOUSE | RuvB-like 2 | 2 | -0.496 | 3.13E-01 | |
| DEK_MOUSE | Protein DEK | 2 | -0.498 | 1.73E-01 | |
| PDIA6_MOUSE | Protein disulfide-isomerase A6 | 7 | -0.501 | 2.10E-01 | |
| RCC2_MOUSE | Protein RCC2 | 2 | -0.502 | 1.60E-01 | |
| PSA1_MOUSE | Proteasome subunit alpha type-1 | 3 | -0.502 | 1.45E-01 | |
| AP1M1_MOUSE | AP-1 complex subunit mu-1 | 2 | -0.508 | 1.06E-01 | |
| CBR1_MOUSE | Carbonyl reductase [NADPH] 1 | 2 | -0.510 | 1.61E-02 | |
| DEST_MOUSE | Destrin | 2 | -0.510 | 6.28E-02 | |
| URP2_MOUSE | Fermitin family homolog 3 | 9 | -0.512 | 2.72E-02 | |
| CATZ_MOUSE | Cathepsin Z | 6 | -0.512 | 4.25E-01 | |
| EFTU_MOUSE | Elongation factor Tu, mitochondrial | 3 | -0.513 | 8.45E-02 | |
| GSLG1_MOUSE | Golgi apparatus protein 1 | 2 | -0.513 | 1.64E-01 | |
| ARPC5_MOUSE | Actin-related protein 2/3 complex subunit 5 | 5 | -0.515 | 2.90E-02 | |
| PP1R7_MOUSE | Protein phosphatase 1 regulatory subunit 7 | 2 | -0.517 | 3.55E-01 | |
| PSB4_MOUSE | Proteasome subunit beta type-4 | 3 | -0.517 | 1.41E-01 | |
| SRSF2_MOUSE | Serine/arginine-rich splicing factor 2 | 2 | -0.519 | 7.47E-02 | |
| VDAC2_MOUSE | Voltage-dependent anion-selective channel protein 2 | 7 | -0.519 | 1.31E-01 | |
| TCPE_MOUSE | T-complex protein 1 subunit epsilon | 8 | -0.522 | 2.84E-02 | |
| ASNA_MOUSE | ATPase Asna1 | 2 | -0.523 | 3.99E-01 | |
| AATM_MOUSE | Aspartate aminotransferase, mitochondrial | 7 | -0.523 | 8.62E-02 | |
| PSMD3_MOUSE | 26S proteasome non-ATPase regulatory subunit 3 | 6 | -0.524 | 2.91E-01 | |
| CHIL3_MOUSE | Chitinase-like protein 3 | 4 | -0.525 | 2.80E-01 | |
| SEPT2_MOUSE | Septin-2 | 5 | -0.525 | 7.63E-02 | |
| ATG7_MOUSE | Ubiquitin-like modifier-activating enzyme ATG7 | 3 | -0.525 | 4.65E-01 | |
| G6PI_MOUSE | Glucose-6-phosphate isomerase | 7 | -0.526 | 1.40E-01 | |
| SNX2_MOUSE | Sorting nexin-2 | 11 | -0.528 | 1.40E-01 | |
| MYO5A_MOUSE | Unconventional myosin-Va | 3 | -0.530 | 2.42E-01 | |
| PDIA3_MOUSE | Protein disulfide-isomerase A3 | 15 | -0.530 | 4.25E-02 | |
| VIME_MOUSE | Vimentin | 28 | -0.537 | 6.49E-02 | |
| TWF2_MOUSE | Twinfilin-2 | 2 | -0.538 | 1.04E-01 | |
| EIF3A_MOUSE | Eukaryotic translation initiation factor 3 subunit A | 13 | -0.539 | 2.17E-01 | |
| CLH1_MOUSE | Clathrin heavy chain 1 | 34 | -0.540 | 4.95E-02 | |
| MDHM_MOUSE | Malate dehydrogenase, mitochondrial | 12 | -0.544 | 1.81E-02 | |
| VATB2_MOUSE | V-type proton ATPase subunit B, brain isoform | 7 | -0.544 | 5.56E-02 | |
| IQGA1_MOUSE | Ras GTPase-activating-like protein IQGAP1 | 40 | -0.545 | 3.25E-02 | |
| MIC60_MOUSE | MICOS complex subunit Mic60 | 6 | -0.546 | 2.61E-01 | |
| ANXA3_MOUSE | Annexin A3 | 8 | -0.548 | 6.09E-02 | |
| NUDC_MOUSE | Nuclear migration protein nudC | 3 | -0.551 | 2.50E-01 | |
| H14_MOUSE | Histone H1.4 | 6 | -0.557 | 1.68E-01 | |
| PCKGM_MOUSE | Phosphoenolpyruvate carboxykinase [GTP], mitochondrial | 2 | -0.559 | 5.97E-02 | |
| PCBP1_MOUSE | Poly(rC)-binding protein 1 | 7 | -0.560 | 1.47E-01 | |
| ANXA5_MOUSE | Annexin A5 | 12 | -0.563 | 5.98E-02 | |
| 1433F_MOUSE | 14-3-3 protein eta | 9 | -0.566 | 9.81E-02 | |
| ATP5H_MOUSE | ATP synthase subunit d, mitochondrial | 3 | -0.569 | 2.30E-01 | |
| RAB2A_MOUSE | Ras-related protein Rab-2A | 4 | -0.570 | 6.10E-02 | |
| HYOU1_MOUSE | Hypoxia up-regulated protein 1 | 9 | -0.571 | 1.55E-01 | |
| ELMO1_MOUSE | Engulfment and cell motility protein 1 | 5 | -0.573 | 1.18E-01 | |
| ERP29_MOUSE | Endoplasmic reticulum resident protein 29 | 4 | -0.575 | 1.20E-02 | |
| PHB_MOUSE | Prohibitin | 5 | -0.576 | 3.37E-03 | |
| HNRPU_MOUSE | Heterogeneous nuclear ribonucleoprotein U | 11 | -0.578 | 5.59E-02 | |
| UD17C_MOUSE | UDP-glucuronosyltransferase 1-7C | 5 | -0.578 | 2.04E-01 | |
| AL5AP_MOUSE | Arachidonate 5-lipoxygenase-activating protein | 2 | -0.578 | 1.32E-01 | |
| ANXA4_MOUSE | Annexin A4 | 14 | -0.579 | 3.14E-02 | |
| THIM_MOUSE | 3-ketoacyl-CoA thiolase, mitochondrial | 4 | -0.579 | 1.08E-01 | |
| TALDO_MOUSE | Transaldolase | 10 | -0.580 | 1.90E-01 | |
| COPG1_MOUSE | Coatomer subunit gamma-1 | 3 | -0.582 | 1.78E-01 | |
| NB5R3_MOUSE | NADH-cytochrome b5 reductase 3 | 5 | -0.583 | 1.42E-01 | |
| NCPR_MOUSE | NADPH--cytochrome P450 reductase | 8 | -0.584 | 2.65E-01 | |
| STIP1_MOUSE | Stress-induced-phosphoprotein 1 | 8 | -0.585 | 6.27E-02 | |
| RS14_MOUSE | 40S ribosomal protein S14 | 5 | -0.585 | 1.86E-01 | |
| PDIA1_MOUSE | Protein disulfide-isomerase | 14 | -0.590 | 1.15E-01 | |
| PKHO2_MOUSE | Pleckstrin homology domain-containing family O member 2 | 3 | -0.592 | 1.86E-01 | |
| COF1_MOUSE | Cofilin-1 | 7 | -0.593 | 9.25E-02 | |
| TPM3_MOUSE | Tropomyosin alpha-3 chain | 6 | -0.595 | 5.45E-02 | |
| DLDH_MOUSE | Dihydrolipoyl dehydrogenase, mitochondrial | 3 | -0.597 | 2.97E-02 | |
| LMNA_MOUSE | Prelamin-A/C | 23 | -0.602 | 6.76E-02 | |
| DHB11_MOUSE | Estradiol 17-beta-dehydrogenase 11 | 2 | -0.602 | 1.82E-02 | |
| DHE3_MOUSE | Glutamate dehydrogenase 1, mitochondrial | 15 | -0.602 | 9.26E-02 | |
| GBG2_MOUSE | Guanine nucleotide-binding protein G(I)/G(S)/G(O) subunit gamma-2 | 2 | -0.604 | 7.76E-02 | |
| KCY_MOUSE | UMP-CMP kinase | 4 | -0.604 | 1.08E-02 | |
| FMNL1_MOUSE | Formin-like protein 1 | 7 | -0.606 | 2.50E-01 | |
| COR1A_MOUSE | Coronin-1A | 9 | -0.608 | 2.17E-01 | |
| VPS35_MOUSE | Vacuolar protein sorting-associated protein 35 | 10 | -0.608 | 4.35E-02 | |
| ERF1_MOUSE | Eukaryotic peptide chain release factor subunit 1 | 5 | -0.610 | 2.63E-03 | |
| ESYT1_MOUSE | Extended synaptotagmin-1 | 12 | -0.610 | 2.48E-01 | |
| C5AR1_MOUSE | C5a anaphylatoxin chemotactic receptor 1 | 2 | -0.611 | 3.73E-01 | |
| MPEG1_MOUSE | Macrophage-expressed gene 1 protein | 10 | -0.613 | 1.25E-01 | |
| LMNB1_MOUSE | Lamin-B1 | 8 | -0.616 | 1.32E-01 | |
| FAS_MOUSE | Fatty acid synthase | 9 | -0.619 | 2.54E-01 | |
| TMED9_MOUSE | Transmembrane emp24 domain-containing protein 9 | 3 | -0.620 | 1.00E-02 | |
| AP2B1_MOUSE | AP-2 complex subunit beta | 11 | -0.620 | 7.95E-02 | |
| ATPA_MOUSE | ATP synthase subunit alpha, mitochondrial | 12 | -0.621 | 3.25E-02 | |
| VA0D1_MOUSE | V-type proton ATPase subunit d 1 | 3 | -0.621 | 1.74E-01 | |
| BIN2_MOUSE | Bridging integrator 2 | 2 | -0.623 | 7.42E-02 | |
| 1433T_MOUSE | 14-3-3 protein theta | 8 | -0.624 | 1.51E-01 | |
| USO1_MOUSE | General vesicular transport factor p115 | 9 | -0.624 | 1.06E-02 | |
| HNRPD_MOUSE | Heterogeneous nuclear ribonucleoprotein D0 | 3 | -0.627 | 2.78E-02 | |
| VPP1_MOUSE | V-type proton ATPase 116 kDa subunit a isoform 1 | 3 | -0.630 | 6.09E-03 | |
| QCR2_MOUSE | Cytochrome b-c1 complex subunit 2, mitochondrial | 2 | -0.633 | 2.00E-01 | |
| SNX5_MOUSE | Sorting nexin-5 | 9 | -0.634 | 2.40E-02 | |
| DHB4_MOUSE | Peroxisomal multifunctional enzyme type 2 | 4 | -0.635 | 8.01E-02 | |
| PMM2_MOUSE | Phosphomannomutase 2 | 2 | -0.638 | 4.98E-02 | |
| PCNA_MOUSE | Proliferating cell nuclear antigen | 7 | -0.640 | 3.07E-01 | |
| SYLC_MOUSE | Leucine--tRNA ligase, cytoplasmic | 4 | -0.642 | 1.66E-02 | |
| MDHC_MOUSE | Malate dehydrogenase, cytoplasmic | 7 | -0.642 | 1.29E-02 | |
| SYNC_MOUSE | Asparagine--tRNA ligase, cytoplasmic | 7 | -0.642 | 1.68E-02 | |
| PAK2_MOUSE | Serine/threonine-protein kinase PAK 2 | 3 | -0.646 | 1.93E-01 | |
| MPU1_MOUSE | Mannose-P-dolichol utilization defect 1 protein | 2 | -0.647 | 7.72E-01 | |
| GBB1_MOUSE | Guanine nucleotide-binding protein G(I)/G(S)/G(T) subunit beta-1 | 4 | -0.649 | 9.40E-02 | |
| CH60_MOUSE | 60 kDa heat shock protein, mitochondrial | 11 | -0.651 | 5.98E-02 | |
| ANXA2_MOUSE | Annexin A2 | 13 | -0.652 | 4.24E-02 | |
| UB2V1_MOUSE | Ubiquitin-conjugating enzyme E2 variant 1 | 2 | -0.653 | 5.10E-02 | |
| IMPA1_MOUSE | Inositol monophosphatase 1 | 2 | -0.654 | 1.34E-01 | |
| IF2G_MOUSE | Eukaryotic translation initiation factor 2 subunit 3, X-linked | 6 | -0.654 | 9.91E-02 | |
| USMG5_MOUSE | Up-regulated during skeletal muscle growth protein 5 | 2 | -0.658 | 1.12E-02 | |
| GMIP_MOUSE | GEM-interacting protein | 2 | -0.660 | 8.85E-02 | |
| GPNMB_MOUSE | Transmembrane glycoprotein NMB | 4 | -0.663 | 1.74E-01 | |
| ANXA1_MOUSE | Annexin A1 | 14 | -0.664 | 6.04E-02 | |
| SP100_MOUSE | Nuclear autoantigen Sp-100 | 2 | -0.664 | 1.14E-01 | |
| HEXB_MOUSE | Beta-hexosaminidase subunit beta | 5 | -0.666 | 1.04E-01 | |
| ASAH1_MOUSE | Acid ceramidase | 6 | -0.666 | 1.57E-01 | |
| CO4B_MOUSE | Complement C4-B | 3 | -0.667 | 2.07E-02 | |
| ITB2_MOUSE | Integrin beta-2 | 12 | -0.671 | 2.66E-02 | |
| GDIB_MOUSE | Rab GDP dissociation inhibitor beta | 14 | -0.676 | 5.78E-02 | |
| OSBL8_MOUSE | Oxysterol-binding protein-related protein 8 | 6 | -0.679 | 4.33E-03 | |
| SAMH1_MOUSE | Deoxynucleoside triphosphate triphosphohydrolase SAMHD1 | 8 | -0.686 | 9.29E-02 | |
| APOBR_MOUSE | Apolipoprotein B receptor | 4 | -0.688 | 1.30E-02 | |
| PLXB2_MOUSE | Plexin-B2 | 2 | -0.689 | 7.24E-02 | |
| FCGR1_MOUSE | High affinity immunoglobulin gamma Fc receptor I | 2 | -0.693 | 3.79E-02 | |
| H2AY_MOUSE | Core histone macro-H2A.1 | 3 | -0.695 | 3.69E-02 | |
| CALU_MOUSE | Calumenin | 2 | -0.697 | 1.62E-02 | |
| IST1_MOUSE | IST1 homolog | 2 | -0.697 | 1.72E-01 | |
| AP1G1_MOUSE | AP-1 complex subunit gamma-1 | 3 | -0.699 | 1.02E-01 | |
| COR1C_MOUSE | Coronin-1C | 3 | -0.699 | 1.39E-01 | |
| TPD54_MOUSE | Tumor protein D54 | 2 | -0.699 | 6.88E-02 | |
| VAMP3_MOUSE | Vesicle-associated membrane protein 3 | 2 | -0.704 | 9.36E-02 | |
| GLTP_MOUSE | Glycolipid transfer protein | 2 | -0.706 | 1.41E-01 | |
| HP1B3_MOUSE | Heterochromatin protein 1-binding protein 3 | 6 | -0.706 | 1.66E-01 | |
| MYO1E_MOUSE | Unconventional myosin-Ie | 10 | -0.709 | 7.29E-02 | |
| VMA5A_MOUSE | von Willebrand factor A domain-containing protein 5A | 16 | -0.710 | 1.17E-01 | |
| AP2A2_MOUSE | AP-2 complex subunit alpha-2 | 3 | -0.711 | 9.54E-02 | |
| BASP1_MOUSE | Brain acid soluble protein 1 | 3 | -0.714 | 1.04E-01 | |
| GANAB_MOUSE | Neutral alpha-glucosidase AB | 3 | -0.714 | 6.51E-02 | |
| CNDP2_MOUSE | Cytosolic non-specific dipeptidase | 11 | -0.715 | 3.49E-02 | |
| CATD_MOUSE | Cathepsin D | 6 | -0.717 | 2.58E-03 | |
| ALDR_MOUSE | Aldose reductase | 8 | -0.726 | 2.11E-03 | |
| PSD12_MOUSE | 26S proteasome non-ATPase regulatory subunit 12 | 4 | -0.735 | 3.07E-02 | |
| GELS_MOUSE | Gelsolin | 11 | -0.739 | 4.69E-02 | |
| AP1B1_MOUSE | AP-1 complex subunit beta-1 | 3 | -0.739 | 2.64E-02 | |
| SPB6_MOUSE | Serpin B6 | 11 | -0.745 | 4.20E-02 | |
| ARC1B_MOUSE | Actin-related protein 2/3 complex subunit 1B | 6 | -0.752 | 2.80E-02 | |
| ARL8A_MOUSE | ADP-ribosylation factor-like protein 8A | 2 | -0.762 | 3.13E-02 | |
| AMPN_MOUSE | Aminopeptidase N | 19 | -0.773 | 1.70E-01 | |
| CASP3_MOUSE | Caspase-3 | 2 | -0.775 | 8.42E-02 | |
| DYN2_MOUSE | Dynamin-2 | 6 | -0.782 | 4.44E-03 | |
| PACN2_MOUSE | Protein kinase C and casein kinase substrate in neurons protein 2 | 2 | -0.783 | 1.09E-01 | |
| H3C_MOUSE | Histone H3.3C | 3 | -0.785 | 1.34E-01 | |
| ABD12_MOUSE | Monoacylglycerol lipase ABHD12 | 2 | -0.797 | 5.03E-01 | |
| HNRPM_MOUSE | Heterogeneous nuclear ribonucleoprotein M | 8 | -0.803 | 6.10E-02 | |
| EHD4_MOUSE | EH domain-containing protein 4 | 14 | -0.811 | 1.58E-05 | |
| LG3BP_MOUSE | Galectin-3-binding protein | 6 | -0.813 | 1.26E-01 | |
| PLCG2_MOUSE | 1-phosphatidylinositol 4,5-bisphosphate phosphodiesterase gamma-2 | 3 | -0.814 | 1.22E-01 | |
| CATS_MOUSE | Cathepsin S | 4 | -0.817 | 7.78E-01 | |
| PLBL2_MOUSE | Putative phospholipase B-like 2 | 2 | -0.821 | 2.29E-01 | |
| VATG1_MOUSE | V-type proton ATPase subunit G 1 | 2 | -0.822 | 6.24E-02 | |
| CAN2_MOUSE | Calpain-2 catalytic subunit | 8 | -0.822 | 1.51E-02 | |
| DBNL_MOUSE | Drebrin-like protein | 2 | -0.823 | 4.93E-02 | |
| ALDH2_MOUSE | Aldehyde dehydrogenase, mitochondrial | 14 | -0.826 | 2.95E-02 | |
| SYHC_MOUSE | Histidine--tRNA ligase, cytoplasmic | 4 | -0.831 | 3.90E-02 | |
| ECHA_MOUSE | Trifunctional enzyme subunit alpha, mitochondrial | 7 | -0.837 | 1.75E-03 | |
| PLD3_MOUSE | Phospholipase D3 | 3 | -0.842 | 7.59E-02 | |
| KCRB_MOUSE | Creatine kinase B-type | 8 | -0.843 | 9.61E-03 | |
| LPXN_MOUSE | Leupaxin | 2 | -0.848 | 7.88E-02 | |
| ASPH_MOUSE | Aspartyl/asparaginyl beta-hydroxylase | 5 | -0.852 | 2.68E-02 | |
| MCM4_MOUSE | DNA replication licensing factor MCM4 | 2 | -0.857 | 1.12E-02 | |
| FUBP2_MOUSE | Far upstream element-binding protein 2 | 2 | -0.860 | 6.05E-02 | |
| DPP3_MOUSE | Dipeptidyl peptidase 3 | 4 | -0.866 | 5.21E-02 | |
| GNAI2_MOUSE | Guanine nucleotide-binding protein G(i) subunit alpha-2 | 9 | -0.868 | 1.02E-01 | |
| APOE_MOUSE | Apolipoprotein E | 2 | -0.869 | 1.04E-01 | |
| FEN1_MOUSE | Flap endonuclease 1 | 2 | -0.870 | 2.04E-02 | |
| SRSF7_MOUSE | Serine/arginine-rich splicing factor 7 | 4 | -0.874 | 1.03E-01 | |
| RMXL1_MOUSE | RNA binding motif protein, X-linked-like-1 | 5 | -0.877 | 9.14E-02 | |
| ODPA_MOUSE | Pyruvate dehydrogenase E1 component subunit alpha, somatic form, mitochondrial | 2 | -0.879 | 4.14E-02 | |
| COX2_MOUSE | Cytochrome c oxidase subunit 2 | 2 | -0.885 | 3.42E-01 | |
| SRRT_MOUSE | Serrate RNA effector molecule homolog | 2 | -0.888 | 1.26E-01 | |
| NCEH1_MOUSE | Neutral cholesterol ester hydrolase 1 | 4 | -0.926 | 3.71E-03 | |
| LSP1_MOUSE | Lymphocyte-specific protein 1 | 5 | -0.930 | 1.06E-02 | |
| MYOF_MOUSE | Myoferlin | 6 | -0.932 | 4.41E-02 | |
| PHB2_MOUSE | Prohibitin-2 | 3 | -0.935 | 2.66E-02 | |
| NUCB1_MOUSE | Nucleobindin-1 | 4 | -0.939 | 7.35E-02 | |
| CPNE3_MOUSE | Copine-3 | 4 | -0.940 | 2.87E-02 | |
| RB11B_MOUSE | Ras-related protein Rab-11B | 7 | -0.942 | 4.40E-02 | |
| LYZ2_MOUSE | Lysozyme C-2 | 3 | -0.946 | 1.03E-02 | |
| CD68_MOUSE | Macrosialin | 2 | -0.946 | 2.28E-02 | |
| ACON_MOUSE | Aconitate hydratase, mitochondrial | 10 | -0.949 | 8.89E-03 | |
| AT2B1_MOUSE | Plasma membrane calcium-transporting ATPase 1 | 3 | -0.958 | 2.91E-01 | |
| STT3A_MOUSE | Dolichyl-diphosphooligosaccharide--protein glycosyltransferase subunit STT3A | 5 | -0.961 | 3.80E-02 | |
| S61A1_MOUSE | Protein transport protein Sec61 subunit alpha isoform 1 | 3 | -0.991 | 6.89E-02 | |
| TRPV2_MOUSE | Transient receptor potential cation channel subfamily V member 2 | 4 | -1.004 | 7.63E-02 | |
| PSD13_MOUSE | 26S proteasome non-ATPase regulatory subunit 13 | 8 | -1.018 | 1.93E-01 | |
| AL9A1_MOUSE | 4-trimethylaminobutyraldehyde dehydrogenase | 9 | -1.025 | 6.07E-02 | |
| SAP_MOUSE | Prosaposin | 9 | -1.027 | 5.80E-02 | |
| DHX58_MOUSE | Probable ATP-dependent RNA helicase DHX58 | 2 | -1.052 | 5.39E-01 | |
| MA2B1_MOUSE | Lysosomal alpha-mannosidase | 2 | -1.059 | 1.12E-01 | |
| SORCN_MOUSE | Sorcin OS=Mus musculus GN=Sri PE=1 SV=1 | 3 | -1.070 | 6.57E-02 | |
| EEA1_MOUSE | Early endosome antigen 1 | 6 | -1.082 | 1.61E-01 | |
| PUR9_MOUSE | Bifunctional purine biosynthesis protein PURH | 8 | -1.133 | 9.01E-02 | |
| BZW1_MOUSE | Basic leucine zipper and W2 domain-containing protein 1 | 3 | -1.138 | 8.72E-02 | |
| SH3L1_MOUSE | SH3 domain-binding glutamic acid-rich-like protein | 2 | -1.254 | 5.72E-01 | |
| PKN1_MOUSE | Serine/threonine-protein kinase N1 | 3 | -1.510 | 5.76E-02 | |
| HEXA_MOUSE | Beta-hexosaminidase subunit alpha | 5 | -1.595 | 2.18E-01 | |

**REFERENCES**

1. Bouquet J, Soloski MJ, Swei A, Cheadle C, Federman S, Billaud JN, et al. Longitudinal Transcriptome Analysis Reveals a Sustained Differential Gene Expression Signature in Patients Treated for Acute Lyme Disease. MBio. 2016;7(1):e00100-16.

2. Gautam A, Dixit S, Philipp MT, Singh SR, Morici LA, Kaushal D, et al. Interleukin-10 alters effector functions of multiple genes induced by *Borrelia burgdorferi* in macrophages to regulate Lyme disease inflammation. Infect Immun. 2011;79(12):4876-92.
